# Inhibition of P2X4R attenuates white matter injury in mice after intracerebral hemorrhage by regulating microglial phenotypes

**DOI:** 10.1186/s12974-021-02239-3

**Published:** 2021-08-23

**Authors:** Xiongjie Fu, Guoyang Zhou, Xinyan Wu, Chaoran Xu, Hang Zhou, Jianfeng Zhuang, Yucong Peng, Yang Cao, Hanhai Zeng, Yin Li, Jianru Li, Liansheng Gao, Gao Chen, Lin Wang, Feng Yan

**Affiliations:** Department of Neurosurgery, Second Affiliated Hospital, School of Medicine, Zhejiang University, Jiefang Road 88th, Hangzhou, 310016 China

**Keywords:** P2X4R, White matter injury, Intracerebral hemorrhage, Microglia polarization, BDNF, TrkB

## Abstract

**Background:**

White matter injury (WMI) is a major neuropathological event associated with intracerebral hemorrhage (ICH). P2X purinoreceptor 4 (P2X4R) is a member of the P2X purine receptor family, which plays a crucial role in regulating WMI and neuroinflammation in central nervous system (CNS) diseases. Our study investigated the role of P2X4R in the WMI and the inflammatory response in mice, as well as the possible mechanism of action after ICH.

**Methods:**

ICH was induced in mice via collagenase injection. Mice were treated with 5-BDBD and ANA-12 to inhibit P2X4R and tropomyosin-related kinase receptor B (TrkB), respectively. Immunostaining and quantitative polymerase chain reaction (qPCR) were performed to detect microglial phenotypes after the inhibition of P2X4R. Western blots (WB) and immunostaining were used to examine WMI and the underlying molecular mechanisms. Cylinder, corner turn, wire hanging, and forelimb placement tests were conducted to evaluate neurobehavioral function.

**Results:**

After ICH, the protein levels of P2X4R were upregulated, especially on day 7 after ICH, and were mainly located in the microglia. The inhibition of P2X4R via 5-BDBD promoted neurofunctional recovery after ICH as well as the transformation of the pro-inflammatory microglia induced by ICH into an anti-inflammatory phenotype, and attenuated ICH-induced WMI. Furthermore, we found that TrkB blockage can reverse the protective effects of WMI as well as neuroprotection after 5-BDBD treatment. This result indicates that P2X4R plays a crucial role in regulating WMI and neuroinflammation and that P2X4R inhibition may benefit patients with ICH.

**Conclusions:**

Our results demonstrated that P2X4R contributes to WMI by polarizing microglia into a pro-inflammatory phenotype after ICH. Furthermore, the inhibition of P2X4R promoted pro-inflammatory microglia polarization into an anti-inflammatory phenotype, enhanced brain-derived neurotrophic factor (BDNF) production, and through the BDNF/TrkB pathway, attenuated WMI and improved neurological function. Therefore, the regulation of P2X4R activation may be beneficial for the reducing of ICH-induced brain injury.

**Supplementary Information:**

The online version contains supplementary material available at 10.1186/s12974-021-02239-3.

## Background

ICH is an acute cerebrovascular disease that is associated with a poor outcomes and a high mortality rate, which places a heavy economic burden on society [1, 2]. A series of pathophysiological processes occur after the onset of ICH, including rapid mass effects of hematomas in the brain and secondary injuries such as neuroinflammation, WMI, oxidative stress, and immune cell infiltration [3–7]. Recently, a series of studies have focused on the therapeutic strategies for ameliorating second injuries after the onset of ICH, and this research has aroused wide interest [8, 9]. However, the protective effects of WMI prevention have not yet been elucidated.

Pathophysiological changes after the onset of ICH include primary and secondary injuries, among which WMI has drawn increasing attention in recent years [10–13]. WMI is one of the leading causes of poor outcomes in ICH, through the mechanism of disrupting the signal transmission; ICH tends to occur within the basal ganglia and internal capsule, which contain abundant white matter (WM) fiber tracts [14]. Therefore, it is highly important to improve ICH outcomes by ameliorating WMI.

Microglia are innate immune cells found in the CNS and respond to exogenous stimuli [15]. Accumulated evidence suggests that microglia exhibit two different phenotypes in response to stimulation: pro-inflammatory and anti-inflammatory phenotypes [16]. Pro-inflammatory phenotype microglia tend to secrete pro-inflammatory cytokines and aggravate the brain injuries, while the anti-inflammatory phenotype microglia contribute strongly to a protective effect [17, 18]. The protective effect of the anti-inflammatory phenotype has drawn much attention. Previous studies have indicated that BDNF, which supports neuronal survival and growth, is strongly associated with the protective effects of anti-inflammatory phenotype microglia [19].

P2X4R is a member of the P2X purine receptor family, which mainly mediates the inflow of sodium and calcium ions and the outflow of potassium ions. P2X4R is highly expressed in activated microglia and has been extensively studied in neuropathic pain and ischemic stroke [20, 21]. Some reports have suggested that P2X4R blockage can ameliorate pathophysiological processes after the exogenous stimuli [22].

TrkB is a member of the receptor tyrosine kinase (RTK) family, which is instrumental in promoting cell survival, differentiation, neurogenesis, and synaptic plasticity. TrkB activation can ameliorate the brain injuries after the onset of ischemic stroke [23, 24], and previous studies have shown that TrkB activation exerts an anti-inflammatory effect in cystitis models as well as antidepressant effects [25, 26]. TrkB has a high affinity for BDNF. BDNF exerts its protective function after exposure to exogenous stimuli through a series of signaling pathways triggered by activated TrkB [27, 28].

However, the potential therapeutic effects of P2X4R after ICH have not yet been elucidated. In the current study, we investigated the protective role of P2X4R inhibition and its effect on polarized microglia, with a focus on anti-inflammatory microglial phenotypes. Underlying mechanisms mediating this association are also discussed here. Namely, we found that the inhibition of P2X4R increased BDNF secretion from anti-inflammatory microglial phenotypes and improved WMI through the BDNF/TrkB pathway.

## Methods

### Animals

Adult C57BL/6N mice (male, 8–10 weeks old, 20–25 g) were purchased from Charles River Laboratory Animal Co., Ltd. (Beijing, China) and were given free access to food and water housed under a 12/12-h dark/light cycle and under specific pathogen-free (SPF) conditions. All procedures in this experiment were performed in accordance with the Guide for the Care and Use of Laboratory Animals published by the National Institutes of Health. All experimental procedures were approved and supervised by the Institutional Ethics Committee of the Second Affiliated Hospital at the Zhejiang University School of Medicine.

### Study design

We conducted the following series of experiments to thoroughly evaluate our study hypotheses. The experiment’s schematic diagram for all experiment is shown in Figure S1.

#### Experiment 1

Forty-five mice were randomly assigned to the following six groups in order to investigate the changes in the protein and expression levels of P2X4R overtime after the onset of ICH: sham (*n* = 8), ICH day 1 (*n* = 5), ICH day 3 (*n* = 8), ICH day 7 (*n* = 11), ICH day 14 (*n* = 8), and ICH day 28 (*n* = 5). Ipsilateral basal ganglia area samples of five mice per group were collected for WB analysis and three mice per group for qPCR. Immunofluorescence staining for P2X4R was performed in the ICH day 7 group (*n* = 3).

#### Experiment 2

Thirty-three mice were randomized into the following three groups to examine WMI after ICH: sham (*n* = 11), ICH day 3 (*n* = 11), and ICH day 7 (*n* = 11). Immunofluorescence staining (*n* = 6 per group) and WB (*n* = 5 per group) were performed to analyze the degree of WMI 3 and 7 days after ICH.

#### Experiment 3

To determine the optimal dose of the P2X4R antagonist 5-BDBD *in vivo*, 12 mice were randomly assigned into the following four groups: ICH + vehicle, ICH + 5-BDBD (0.3 mg/kg), ICH + 5-BDBD (1 mg/kg), and ICH + 5-BDBD (3 mg/kg) (*n* = 3, per group). Hematoma basal ganglia samples from three mice in each group were collected for WB analysis.

#### Experiment 4

Seventy-eight mice were divided randomly into six groups to explore the impact of P2X4R on the microglial phenotypes, WMI, and the change of P2X7R after ICH: sham day 3 (*n* = 8), sham day 7 (*n* = 8), ICH + vehicle day 3 (*n* = 13), ICH + vehicle day 7 (*n* = 13), ICH + 5-BDBD day 3 (*n* = 13), and ICH + 5-BDBD day 7 (*n* = 13) groups. The ICH + vehicle group mice were administered the vehicle used for 5-BDBD after ICH. The ICH + 5-BDBD group was treated with an optimal 5-BDBD dose after ICH. Immunofluorescence (*n* = 5 per group) and qPCR (*n* = 3 per group) were performed within the six groups to analyze the status of microglia at 3 and 7 days after ICH with 5-BDBD treatment. Subsequently, we performed immunofluorescence staining (*n* = 5) and WB (*n* = 5 per group) to detect the WMI with 5-BDBD treatment after ICH. qPCR and WB were performed to analyze the protein and expression levels of P2X purinoreceptor 7 (P2X7R) with 5-BDBD treatment after ICH.

#### Experiment 5

To explore the effects of inhibited P2X4R on neurofunction after ICH, 16 mice were randomly divided into two groups for the following experiment. Neurological scores were evaluated in each group of mice at 1, 3, 7, 14, and 28 days after ICH (*n* = 8, per group).

#### Experiment 6

To investigate the protective effect of P2X4R inhibition and the potential underlying mechanisms after ICH, Twelve mice were divided randomly into two groups: ICH + 5-BDBD and ICH + 5-BDBD + ANA-12. Mice in the ICH + 5-BDBD group were treated with 5-BDBD after ICH induction. The ICH + 5-BDBD + ANA-12 group received ANA-12 injections abdominally following the 5-BDBD treatment. The mice underwent corner turn test, cylinder test, forelimb placing test, and wire hanging test at 1, 3, 7, 14, and 28 days after ICH to evaluate the neuronal function.

#### Experiment 7

This experiment was conducted to investigate the effects of ANA-12 on WIM after ICH and TrkB activity after ICH. Thirty mice were divided randomly into the following six groups: sham day 3 (*n* = 3), sham day 7 (*n* = 3), ICH + vehicle day 3 (*n* = 6), ICH + vehicle day 7 (*n* = 6), ICH + ANA-12 day 3 (*n* = 6), and ICH + ANA-12 day 7 (*n* = 6). The brains were collected for immunofluorescence staining and WB in order to observe WMI following ANA-12 treatment for ICH on days 3 and 7. Effects of ANA-12 on TrkB activity after ICH were evaluated on day 7.

#### Experiment 8

To further explore the mechanisms underlying the protective effects of P2X4R inhibition, fifty mice were randomly divided into six groups: ICH + vehicle day 3 (*n* = 5), ICH + vehicle day 7 (*n* = 5), ICH + 5-BDBD day 3 (*n* = 10), ICH + 5-BDBD day 7 (*n* = 10), ICH + 5-BDBD + ANA-12 day 3 (*n* = 10), and ICH + 5-BDBD + AND-12 day 7 groups (*n* = 10). The brains were collected for immunofluorescence staining and WB to observe the pathophysiological processes on days 3 and 7 after the onset of ICH via detecting myelin basic protein (MBP) expression levels.

### ICH mouse model

To simulate the pathophysiological progress after the onset of ICH, ICH mouse models were established according to previous studies [1, 29]. Briefly, mice were anesthetized intraperitoneally with pentobarbital sodium (40 mg/kg, 1%). Then, 0.05 U collagenase (type VII, from *Clostridium histolyticum*; Sigma-Aldrich) in 0.5 μl saline was prepared within 1 h and kept in an ice bath; it was injected into the region of the right basal ganglia (2.5 mm lateral to the bregma, 3 mm deep at a 5° angle) stereotactically in 5 min and was followed by another 5 min of waiting in case of reflux. Rectal temperature was monitored throughout the ICH induction and was maintained at 37.0 °C ± 0.5 °C, and the mice in the sham group received the same treatment, including needle insertion, with the exception for collagenase injection.

### WB analysis

Mice were anesthetized and subjected to intracardiac perfusion with 0.1 mol/L phosphate-buffered saline (PBS). The brain was cut into 1-mm thick coronal brain slices, and the tissue of the hematoma basal ganglia regions was collected. The tissue was then homogenized in a RIPA lysis buffer (Beyotime, Shanghai, China) and centrifuged for 15 min at 12,000 *g* at 4 °C. A BCA protein assay kit (Thermo Fisher Scientific, Waltham, MA, USA) was used to determine protein concentrations. WB was performed according to the manufacturer’s instructions [30]. Briefly, the same amounts of protein (40 μg) from each sample were suspended in loading buffer and loaded onto SDS-PAGE gels. The proteins were electrophoresed and electro-transferred onto nitrocellulose membranes. The membranes were blocked with 5% milk at room temperature for 1 h and incubated overnight with the following primary antibodies: P2X4R (1:1000; APR-002; Alomone), MBP (1:1000; CST#83683S; Cell Signaling Technology), amyloid precursor protein (APP) (1:1000; 32136; Abcam), BDNF (1:1000; 28205-1-AP; Proteintech), p-TrkB (1:1000; ABN1381; Millipore), TrkB (1:100; sc-7268; Santa Cruz), P2X7R (1:1000; APR-004; Alomone), and β-actin (1:5000, 8226; Abcam). The membranes were then incubated with horseradish peroxidase-conjugated secondary antibodies (Beyotime, Shanghai, China) for 1 h at room temperature. The ECL Plus Chemiluminescence Reagent Kit (Millipore, WBNLS0500) was used to observe the protein bands, and the densities were detected using ImageJ software (Nation Institutes of Health, Bethesda MA, USA).

### Immunofluorescence staining

Mice were first anesthetized and perfused with 20-mL ice-cold 0.1 mol/L PBS via the cardiac apex, followed by perfusion with 4% paraformaldehyde (PFA). The whole brain was removed and immersed in 4% PFA overnight and was immersed in 30% sucrose for 72 h at 4 °C. Brain samples were cut into coronal slices (10 μm) and fixed onto slides for subsequent experiments. After washing with PBS and incubation with 10% donkey serum containing 0.3% Triton X-100 for 1 h at room temperature, the sections were incubated with primary antibodies overnight at 4 °C, including P2X4R (1:200, Alomone, APR-002), Iba-1 (1:500, Abcam, 5076), NeuN (1:1000, Abcam, 177487), GFAP (1:500, Millipore, MAB360), MBP (1:500, Cell Signaling Technology, CST#83683S), Neurofilament-200 (NF-200) (1:1000, Sigma, N4142), CD16/32 (1:100, Abcam, 24235), and Arg-1(1:500, Proteintech, 16001-1-AP). The cryosections were washed with PBS and incubated with the following secondary antibodies at 37 °C for 1 h: Alexa Fluor 488-conjugated donkey anti-rabbit IgG (Invitrogen, 21206), Alexa Fluor 594-conjugated donkey anti-goat IgG (Invitrogen, 11058), Alexa Fluor 488-conjugated donkey anti-mouse IgG (Invitrogen, 21202), Alexa Fluor 555-conjugated donkey anti-rabbit IgG (Invitrogen, 31572), Alexa Fluor 488-conjugated donkey anti-Rat IgG (Invitrogen, 21208), and Alexa Fluor 488-conjugated donkey anti-goat IgG (Invitrogen, 32814) at 37 °C for 1 h. Finally, brain sections were stained with DAPI (Abcam, ab104135) and were imaged using a fluorescence microscope (Leica, Mannheim, Germany) with a 20× or 40× objective.

Immunostaining images were analyzed in a blinded manner using FIJI software (NHI). Fluorescence intensity was quantified, as described previously [31–33]. In brief, two regions of interest (ROIs) surrounding the hematoma were randomly selected and captured using the same imaging parameters. All images were converted to grayscale and split by color channel. The fluorescence intensity was calculated as the percentage of fluorescence-positive pixels within each region. The final values are expressed as percentage change in the sham group; the 100% was the mean level of white matter in this group. For the colocalization analysis, the NeuN-positive, GFAP-positive, and Iba-1 positive cell were manually counted in each ROI. The individual images of the two labels were merged using FIJI software, and the double-labeled cells were counted manually. The percentages of double-labeling were then calculated. The morphological analysis of microglia was performed according to previous protocols [34, 35]. Three to six mice from each group were analyzed, and at least two images per mouse were analyzed per group. A general schematic diagram of the ROI is shown in Figure S2.

### Assessment of neurobehavioral function

Neurobehavioral function assessments were performed by two researchers blinded to the experimental conditions. Four tests, including the corner turn test, the cylinder test, the forelimb placing test, and the wire hanging test, were conducted to evaluate the neurobehavioral function on days 1, 3, 7, 14, and 28 after the onset of ICH [10, 36]. In the corner turn test, a 30° corner consisting of two plastic walls was prepared for the mice; the mice could turn either right or left to exit the corner freely. The test was repeated 10 times for each mouse, and the percentage of right turns was recorded. For the cylinder test, the mice were provided with a transparent cylinder (diameter: 8 cm; height: 25 cm). They were allowed to rear freely 20 times, and the forelimb that was used to rear against the wall was recorded. Their scores were calculated as follows: (R − L)/(R + L + B); a greater score suggested a more severe neuronal dysfunction. In the forelimb placing test, each mouse was held by its torso and allowed to hang its forelimb freely. Each forelimb was tested 10 times, and the percentage of trials in which the mouse placed the correct forelimb on the countertop in response to the vibrissae stimulation was recorded. In the wire hanging test, the animals were placed on a stainless steel bar with a length of 50 cm and a diameter of 2 mm. The bar was fixed at 37 cm above a flat surface. The mice were tested for 30 s in three trials. The scores were calculated according to the following system: 0 (fell off), 1 (hanging onto the bar with two forepaws), 2 (hung onto the bar with added attempt to climb onto the bar), 3 (hung onto the bar with two forepaws and one or two hind paws), 4 (hung onto the bar with all four paws and with tail wrapped around the bar), and 5 (escaped to one of the supports).

### Drug administration

Drug administration consisted of 5-BDBD, a P2X4R inhibitor, which was purchased from Sigma-Aldrich and diluted to 1.25 mg/mL in 0.5% methylcellulose. The optimal 5-BDBD dose was evaluated (Figure S4). The 5-BDBD at 3 mg/kg/day or an equal volume of vehicle was administered via oral gavage 30 min after ICH and was subsequently administered once daily for 7 consecutive days. ANA-12 was obtained from MedChem Express (Monmouth Junction, NJ, USA) and was diluted in DMSO. The optimal dose of ANA-12 was based on a previous report [25, 37]. The mice received 0.5 mg/kg/day of ANA-12 intraperitoneally. The first injection was performed 1 day before ICH and was subsequently administered daily for 7 days.

### Quantitative polymerase chain reaction (qPCR)

Total RNA from the hematoma basal ganglia was isolated using TRIzol reagent (Invitrogen, Thermo Fisher, MA, USA), according to the manufacturer’s protocol. Complementary deoxyribonucleic acid (cDNA) was synthesized using the PrimeScript^TM^ RT Master Kit (Takara Bio Inc., Shiga, Japan). qPCR was performed using Applied Biosystems Quant Studio ^TM^ 5 software (Thermo Fisher Scientific, Waltham, MA, USA). β-Actin was used as an internal control. The primers used for this process are listed in Table S1.

### Statistical analysis

Data are presented as the means ± standard errors of the mean (SEM). Tests for normal distribution and homogeneity of variance and comparisons between multiple groups were conducted using one-way analysis of variance. Persistent neurological functions were analyzed via two-way repeated-measures ANOVA followed by Tukey’s *post hoc* test. The non-normal distribution and unequal variance parameters were compared using the Kruskal–Wallis test with Bonferroni correction for *post hoc* comparisons. The threshold for significance was set at *P* < 0.05. Statistical analyses were conducted using GraphPad Prism 8.0 (GraphPad Prism Software Inc., San Diego, CA, USA) and SPSS 22.0 for Windows (SPSS, Inc., Chicago, IL, USA).

## Results

### P2X4R expression in the brain increased after ICH

**Fig. 1:**
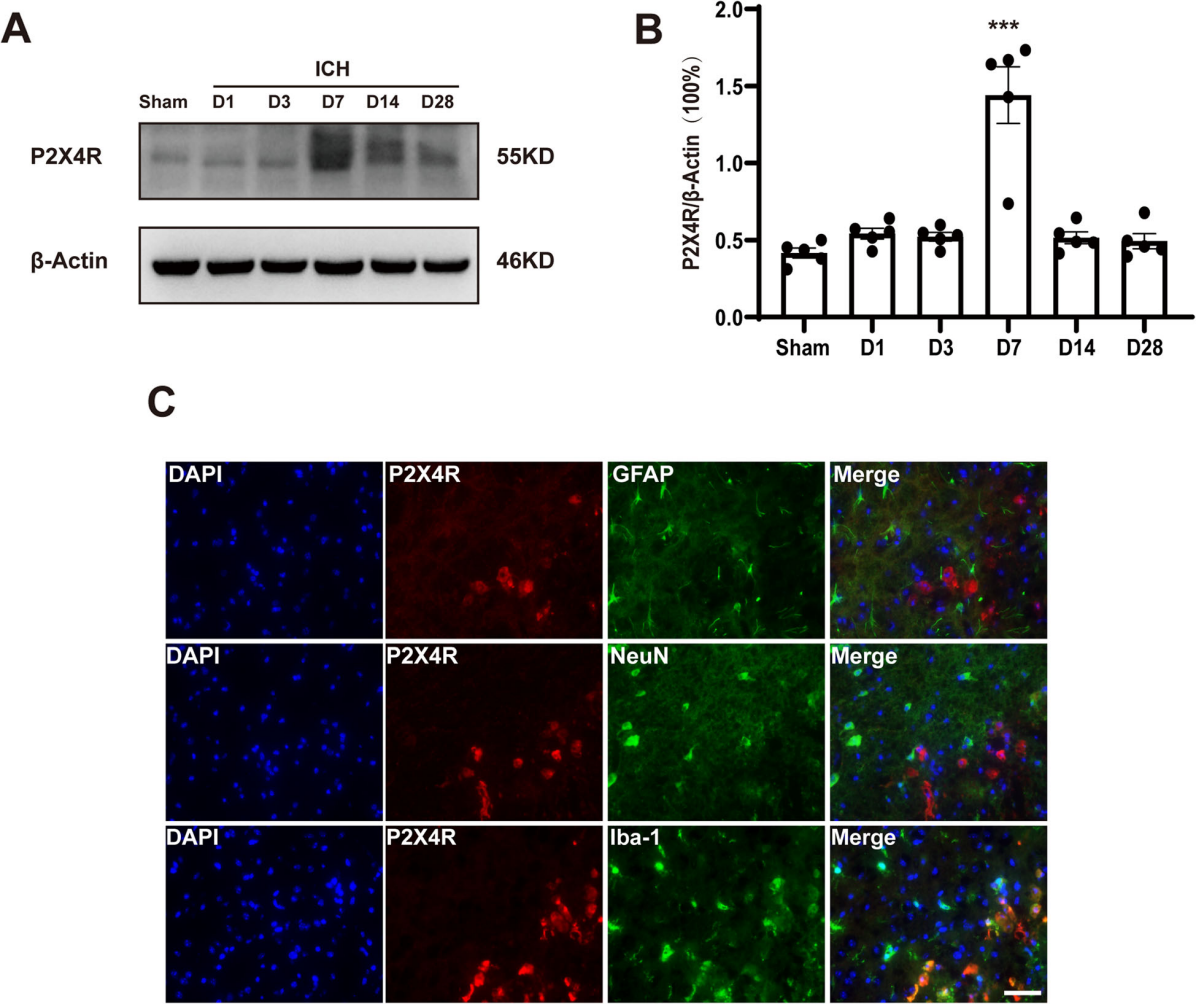
The expression of P2X4R in microglia increased after ICH. A Protein expression of P2X4R was analyzed by western blot in the hematoma hemisphere at different time points after ICH (*n* = 5). B Quantification of P2X4R at the protein level. C Immunofluorescence staining of P2X4R/GFAP, P2X4R/NeuN-1, and P2X4R/ Iba-1 in the murine brain 7 days after ICH (*n* = 3). Data are expressed as the means ± SEM. *P < 0.05. **P < 0.01. ***P < 0.001. Scale bar = 50 μm

To investigate changes in P2X4R after ICH onset, we first explored P2X4R protein expression levels in the brain at different time points after ICH. Our results showed that, after ICH, protein expression was increased compared with that in the sham group. The increasing trend was most evident at 7 days after ICH (Fig. 1A, B). Moreover, P2X4R transcript levels increased after ICH, peaking at 7 days and then began to decrease (Figure S3 A).

We found that co-staining of P2X4R with Iba-1, NeuN, and GFAP revealed that P2X4R was mainly expressed in brain microglia 7 days after the onset of ICH (Fig. 1C, Figure S3 B). This indicates that microglia play a vital role in the P2X4R-mediated brain injury, including WMI.

### White matter damage after induction of ICH

**Fig. 2:**
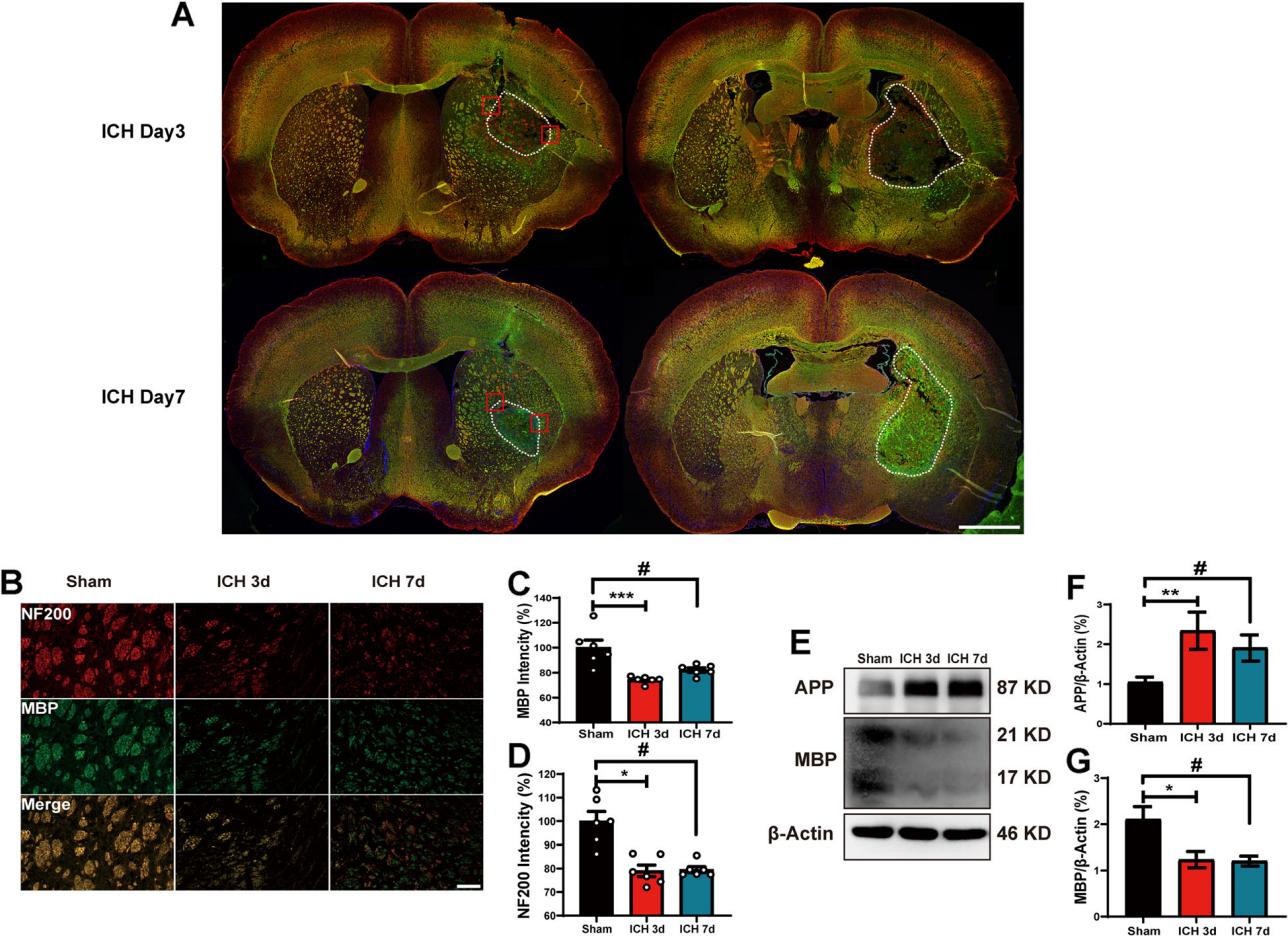
White matter injury after ICH. A Representative image of white matter injury immunostaining after ICH on days 3 and 7. The red box represents the ROI, and the white dotted line represents the hematoma region. B Immunostaining for MBP and NF200 in sham, vehicle, ICH day 3, and ICH day 7 mice. C and D The mean fluorescence densities of MBP and NF200 in the murine brain on days 3 and 7 after ICH (*n* = 6 per group). E–G Western blot shows the protein expression of MBP and APP in the hematoma region (*n* = 5 per group). Data are expressed as the means ± SEM. *P < 0.05. **P < 0.01. ***P < 0.001 vs. ICH day 3 group, ^#^*P* < 0.05. ^##^*P* < 0.01. ^###^*P* < 0.001 vs. ICH day 7 group. Scale bar = 100 μm

We evaluated the integrity of WM with MBP, using immunofluorescence staining and WB in the area of the basal ganglia (Fig. 2A). MBP is a protein that represents an intact myelin sheath. We found that after ICH, the mean fluorescence density of MBP decreased on days 3 and 7, which is consistent with the changes in protein levels (Fig. 2B, C, E, G). Previous studies have shown that APP accumulates after axonal injury, and that APP protein levels increase on days 3 and 7 after ICH (Fig. 2E, F). NF200 was assessed using immunofluorescent staining to detect the condition of axons. Our results showed that fluorescence density decreased significantly compared with that in the sham group (Fig. 2B, D). Our study thus showed that ICH can lead to serious damage to WM.

### Inhibition of P2X4R ameliorated brain injury after ICH

To determine the optimal dose of 5-BDBD needed to inhibit P2X4R, three doses were used: 0.3, 1, and 3 mg/kg. The WB results indicated that a 3-mg/kg dose of 5-BDBD treatment significantly reduced P2X4R protein levels when compared with the other groups (Figure S4 A, B). Based on our results, a dosage of 3 mg/kg was chosen as the optimal dosage of 5-BDBD, and the dosage was used in subsequent experiments.

**Fig. 3:**
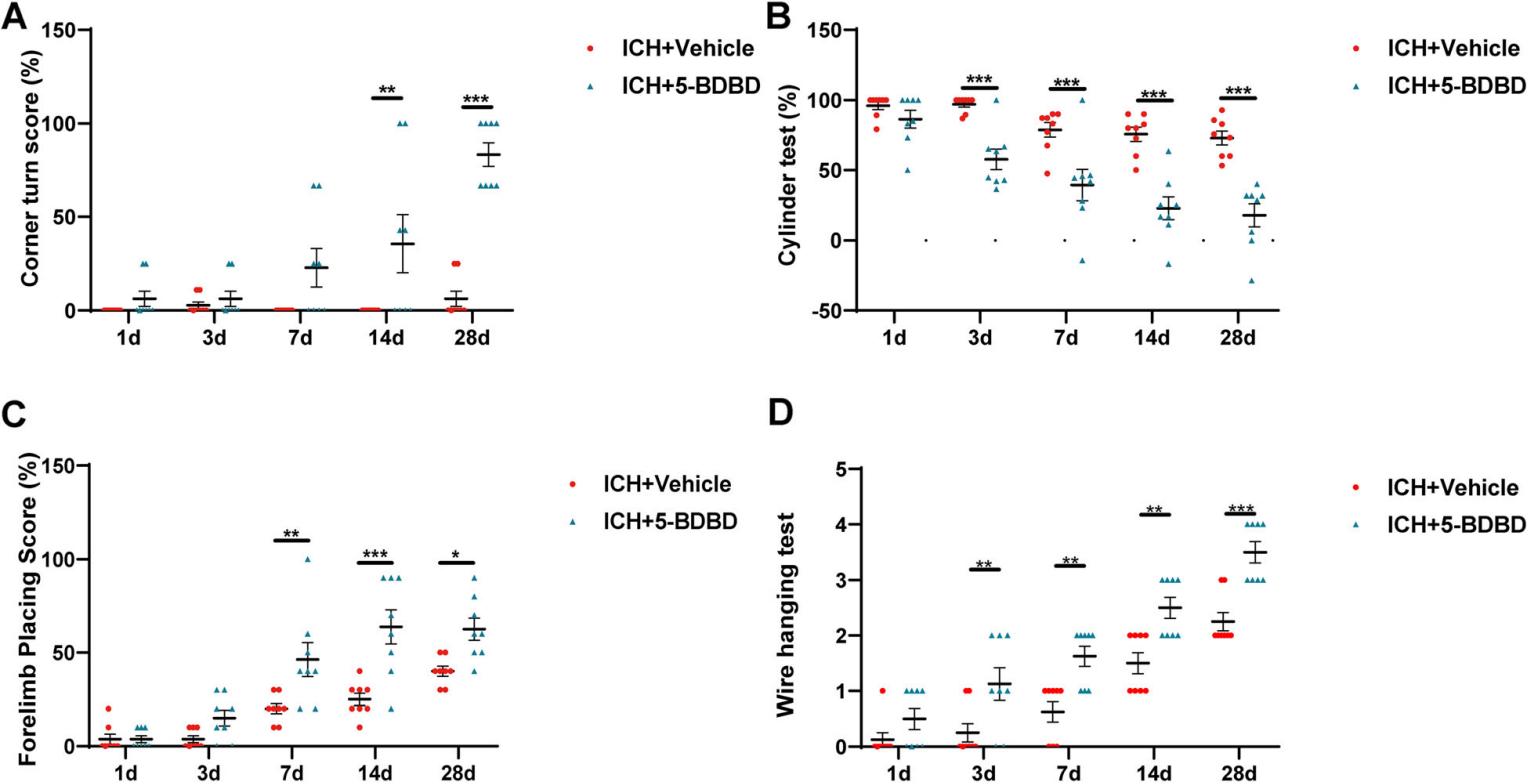
Effects of P2X4R on neurological functions after ICH. A Corner turn test, B cylinder test, C forelimb placing test, D Wire hanging test. (*n* = 8 per group). Data are expressed as the means ± SEM. *P < 0.05. **P < 0.01. ***P < 0.001 vs. ICH + vehicle group

To explore the role of P2X4R in ICH-induced brain injury, we compared neurofunctions between the ICH + vehicle and ICH + 5-BDBD groups. In the corner turn test, the ICH + 5-BDBD group showed a better score than the ICH + vehicle group on days 14 and 28 (*Fig.* *3**A*). In the cylinder test, the ICH + 5-BDBD group displayed a low percentage compared to the ICH + vehicle group on days 3, 7, 14, and 28 (*Fig.* *3**B*). In the forelimb placing test, the ICH + 5-BDBD group showed an obvious improvement in the score for the right forelimbs as compared with the ICH + vehicle group at 7, 14, and 28 days after ICH (*Fig.* *3**C*). In the wire hanging test, the ICH + 5-BDBD group presented a better score than the ICH + vehicle group at 3, 7, 14, and 28 days after ICH (*Fig.* *3**D*).

### Inhibition of P2X4R increases the phenotypic switch of microglia from pro-inflammatory to anti-inflammatory phenotypes

**Fig. 4:**
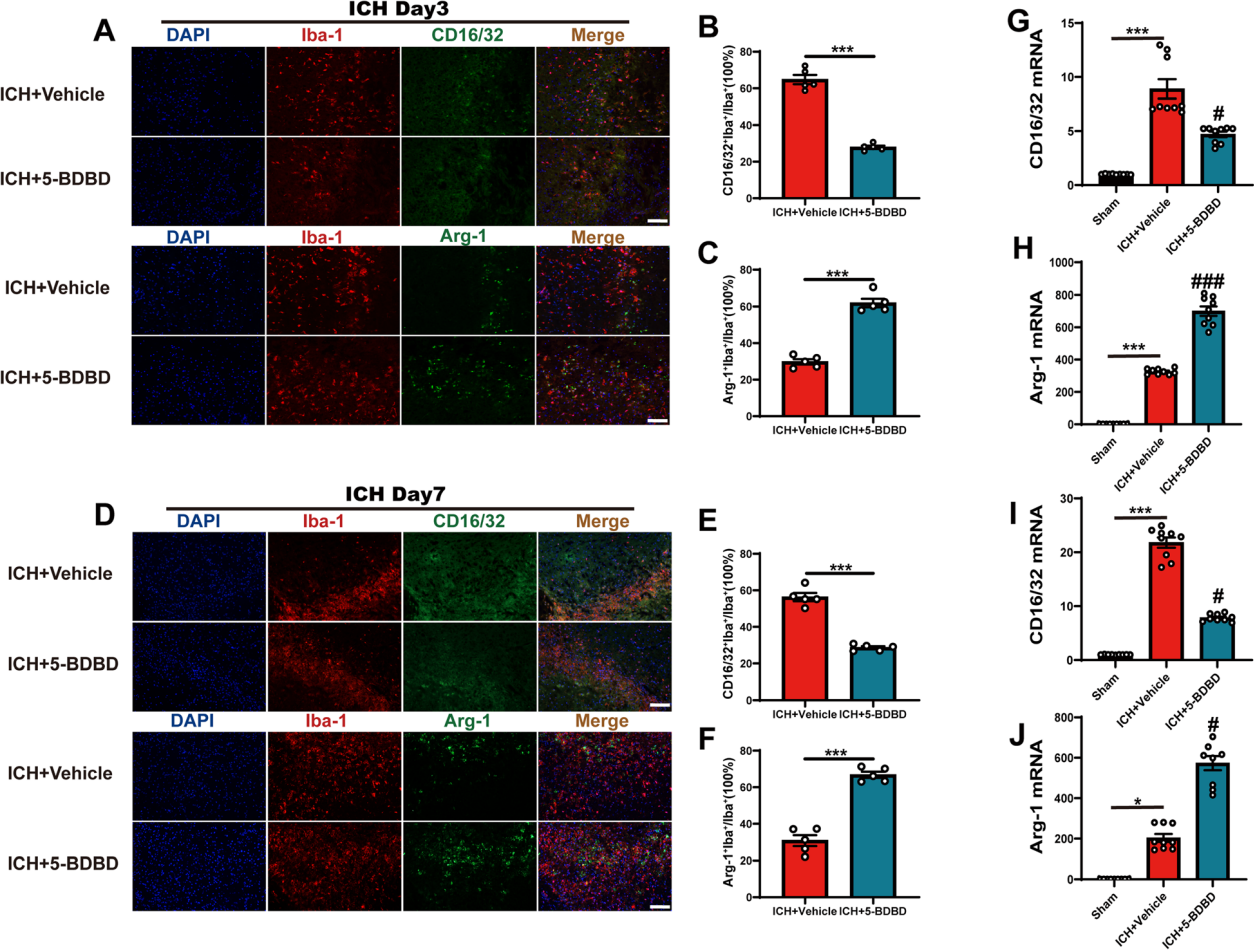
P2X4R blockage promoted the phenotype of microglia from pro-inflammatory into anti-inflammatory after ICH. A–C Immunostaining for CD16/32^+^Iba1^+^/Iba1^+^ and Arg-1^+^Iba1^+^/Iba1^+^ in ICH + vehicle and ICH + 5-BDBD groups after ICH day 3 (*n* = 5 per group). D–F Immunostaining for CD16/32^+^Iba1^+^/Iba1^+^ and Arg-1^+^Iba1^+^/Iba1^+^ in ICH + vehicle and ICH + 5-BDBD groups after ICH day 7 (*n* = 5 per group). G, H Levels of CD16/32 and Arg-1 mRNA transcription were examined by qPCR in the sham, vehicle, and 5-BDBD groups after ICH day 3 (*n* = 3 per group). I, J Relative mRNA expression of CD16/32 and Arg-1 were examined by qPCR in the sham, vehicle, and 5-BDBD groups after ICH day 7 (*n* = 3 per group). Data are expressed as the means ± SEM. *P < 0.05. **P < 0.01. ***P < 0.001 vs. ICH + vehicle group. ^#^*P* < 0.05. ^##^*P* < 0.01. ^###^*P* < 0.001 vs. ICH + 5-BDBD group. Scale bar = 100 μm

To further explore the role of P2X4R in ICH, we considered the contribution of microglia during the pathophysiological process. We investigated the phenotypic switch in microglia using immunofluorescence staining. We found that the ratio of pro-inflammatory phenotype microglia (CD16/32^+^Iba1^+^/Iba1^+^) increased significantly on days 3 and 7 after the induction of ICH, which is consistent with the results of previous studies [38]. Interestingly, after the treatment with 5-BDBD, we found that the activation of pro-inflammatory microglial phenotypes decreased significantly on days 3 and 7 after ICH, while the anti-inflammatory phenotype microglia (Arg-1^+^Iba1^+^/Iba1^+^) increased compared with the ICH + vehicle group (*Fig.* *4**A–F*). In addition, ICH promoted the transformation of microglia into an activated state and hypertrophic morphology with larger soma and short protrusions. After the administration of 5-BDBD, we found that the number of microglia surrounding hematoma regions were reduced and that the soma was decreased and presented with protrusion (Figure S5 C-H).

In addition, we examined changes in the mRNA expression levels of pro-inflammatory and anti-inflammatory marker genes in the hemorrhagic hemisphere; mRNA expression of pro-inflammatory markers, including CD16/32, IL-1β, and TNF-α, was increased at 3 and 7 days after ICH, and this trend was reversed by the 5-BDBD treatment (*Fig.* *4**G, I* and Figure S5 A, B). However, the mRNA expression levels of the anti-inflammatory markers Arg-1 and CD206 were enhanced in the ICH + 5-BDBD group as compared with the ICH + vehicle group after ICH 3 and 7 days after ICH (*Fig.* *4**H, J* and Figure S5 A, B). Our results suggest that P2X4R plays a vital role in the phenotypic switch of microglia after the onset of ICH and that the inhibition of P2X4R may be beneficial in ameliorating ICH-induced neuroinflammation.

Moreover, P2X4R might be able to form heteromers with P2X7R. We examined the protein and mRNA expression levels of P2X7R in ICH mice after the administration of 5-BDB. Our results show that 5-BDBD did not affect P2X7R after ICH (Figure S6 A-C).

### Inhibition of P2X4R can increase the expression level of BDNF in microglia

**Fig. 5:**
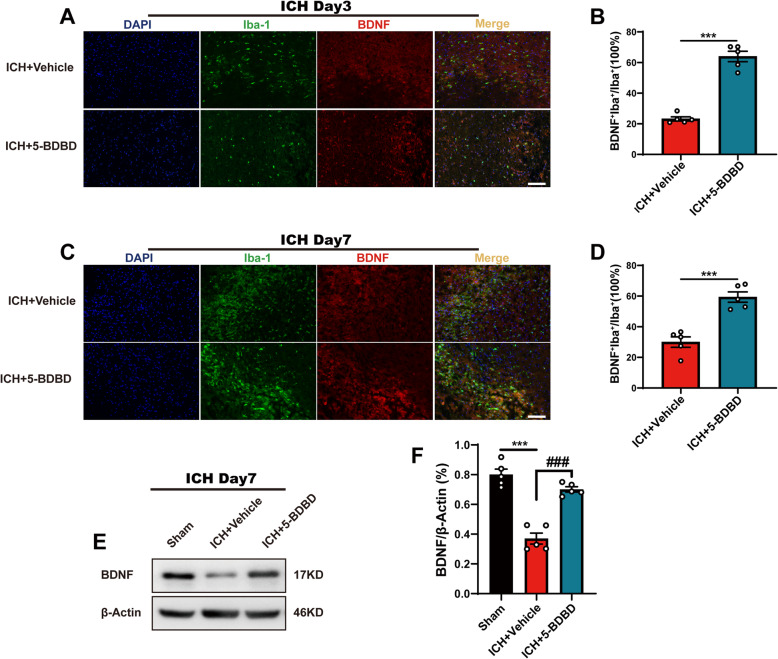
P2X4R inhibition increased BNDF production in microglia after ICH. A, B BNDF immunostaining in the ICH + vehicle and ICH + 5-BDBD groups after ICH day 3 with quantification. (*n* = 5 per group). C, D Immunostaining for BDNF^+^Iba-1^+^/Iba-1^+^ in the ICH + vehicle and ICH + 5-BDBD groups after ICH day 7 (*n* = 5 per group). E, F Western blots show the protein expression of BDNF in the peri-hematoma region with quantification after ICH day 7 (*n* = 5 per group). Data are expressed as the means ± SEM. *P < 0.05. **P < 0.01. ***P < 0.001 vs. ICH + vehicle group. ^#^*P* < 0.05. ^##^*P* < 0.01. ^###^*P* < 0.001 vs. ICH + 5-BDBD group. Scale bar = 100 μm

BDNF is a neuroprotective factor that can support neuronal survival and growth and has a strong connection with the protective effects of anti-inflammatory microglia [19]. Based on the observation that the ratio of anti-inflammatory microglia increased significantly after the administration of 5-BDBD, we found that the number of cells that can secrete BDNF increased significantly after the inhibition of P2X4R by 5-BDBD administration as compared to the ICH + vehicle group (*Fig.* *5**A–D*). We further investigated BDNF protein levels in mice after ICH. The results showed that the protein expression level of BDNF decreased significantly on day 7 after the induction of ICH, while P2X4R inhibition by 5-BDBD reversed this decrease (*Fig.* *5**E, F*). Our study suggests that P2X4R plays an important role in the modulation of BDNF expression in microglia, and can thus be a potential target for ameliorating WMI after ICH.

### Inhibition of P2X4R ameliorates the WMI after ICH

**Fig. 6:**
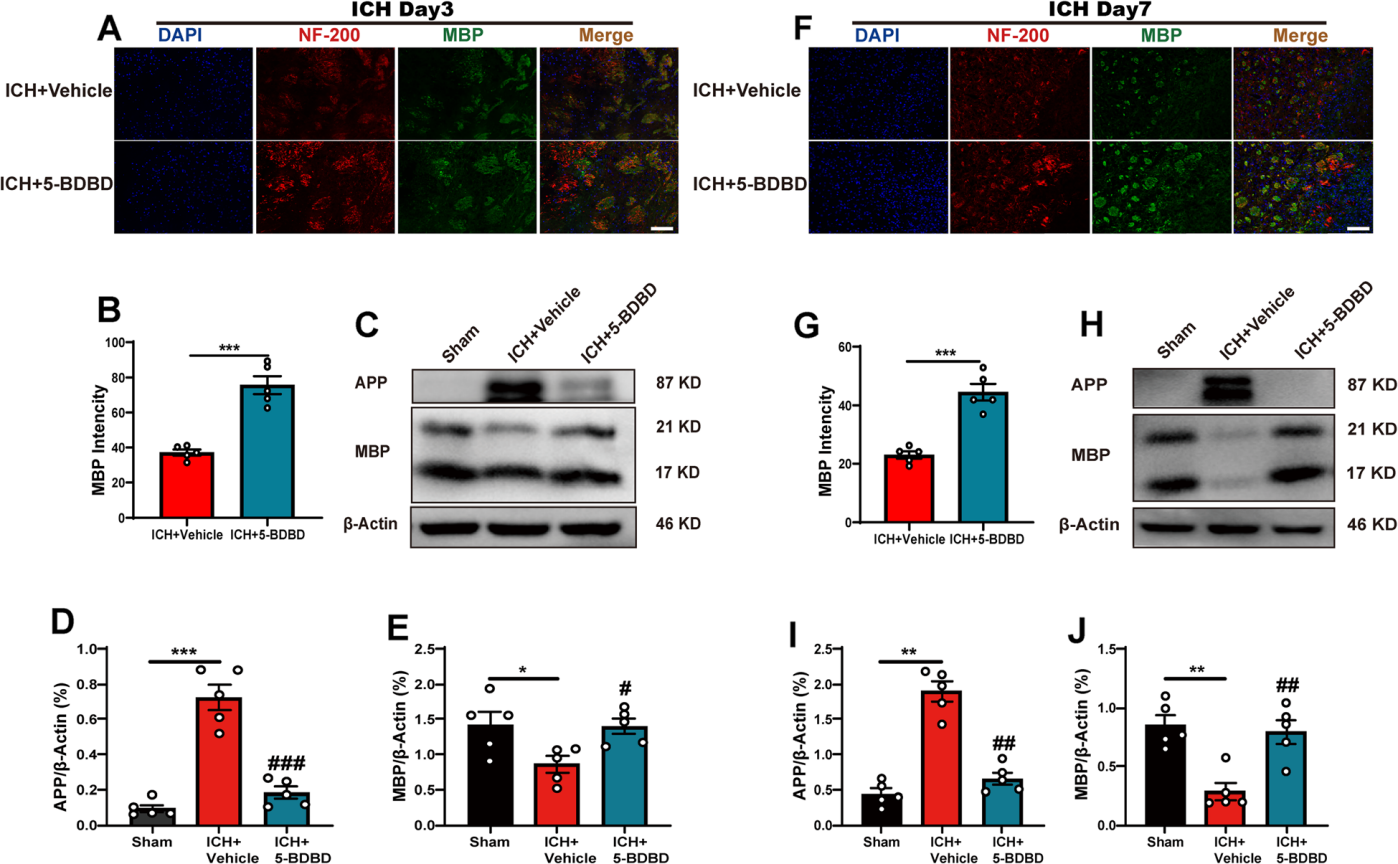
The inhibition of P2X4R alleviates white matter injury after ICH. A Representative images of double immunofluorescence staining for NF200 and MBP in the peri-hematoma region on the third day after ICH. B Mean fluorescence densities of MBP after 3 days of ICH (*n* = 5 per group). C–E Representative western blot images show the protein expression of MBP and APP in the hematoma region in the 5-BDBD treatment groups compared with the vehicle group 3 days after ICH (*n* = 5 per group). F Double immunofluorescence staining for MBP and NF200 in the peri-hematoma region 7 days after ICH. G Mean fluorescence densities of MBP 7 days after ICH (*n* = 5 per group). H–J Representative western blot images show the protein expression of MBP and APP in the hematoma region in the 5-BDBD treatment groups compared with the vehicle group 7 days after ICH (*n* = 5 per group). Data are expressed as the means ± SEM. *P < 0.05. **P < 0.01. ***P < 0.001 vs. ICH + vehicle group. ^#^*P* < 0.05. ^##^*P* < 0.01. ^###^*P* < 0.001 vs. ICH + 5-BDBD group. Scale bar = 100 μm

Our study demonstrated that 5-BDBD can prevent the decrease in the mean fluorescence intensity of MBP in the peri-hematoma region after ICH days 3 and 7 (*Figs.* *6**A, B and* *6**F, G*), showing that the P2X4R inhibitor 5-BDBD attenuated WMI induced by ICH. Our results further showed that the protein level of APP increased significantly, while the MBP decreased at day 3 and day 7 after ICH; this trend was reversed after the inhibition of P2X4R through administration of 5-BDBD (*Figs.* *6**C–E and* *6**H–J*). Our results showed that P2X4R greatly contributed to axonal damage and myelin degradation, while the inhibition of P2X4R can ameliorate WMI.

### Potential mechanism underlying the protective effect of P2X4R inhibition

Our immunofluorescence staining results showed that TrkB was co-stained with Olig2 (Figure S7), which is consistent with the results of previous research. Previous studies have shown that the activation of TrkB can ameliorate the pathophysiological processes in different diseases, such as ischemic stroke and cystitis, and exert an antidepressant effect [23–26]. TrkB is a downstream receptor for BDNF and has a high affinity for binding. ANA-12, an inhibitor of TrkB, has been widely used in many studies. First, we tested the effect of ANA-12 alone on WMI after ICH and the effect of ANA-12 on TrkB activity after ICH. Our results showed that protein levels of MBP decreased at days 3 and 7 after ICH, and treatment with ANA-12 alone did not significantly affect this trend (Figure S8 A-D). In addition, compared with the ICH + vehicle group, the mean fluorescence intensity of MBP did not differ after ANA-12 treatment on days 3 and 7 after ICH (Figure S8 E-H). According to previous research, p-TrkB is the active form of TrkB. Our WB results showed that the protein level of p-TrkB was reduced, and TrkB has not significantly differed after ANA-12 treatment compared with the ICH + vehicle group. The ratio of p-TrkB/TrkB was reduced after ANA-12 treatment (Figure S8 I, J). This result indicated that ANA-12 effectively inhibit TrkB activation after ICH.

**Fig. 7:**
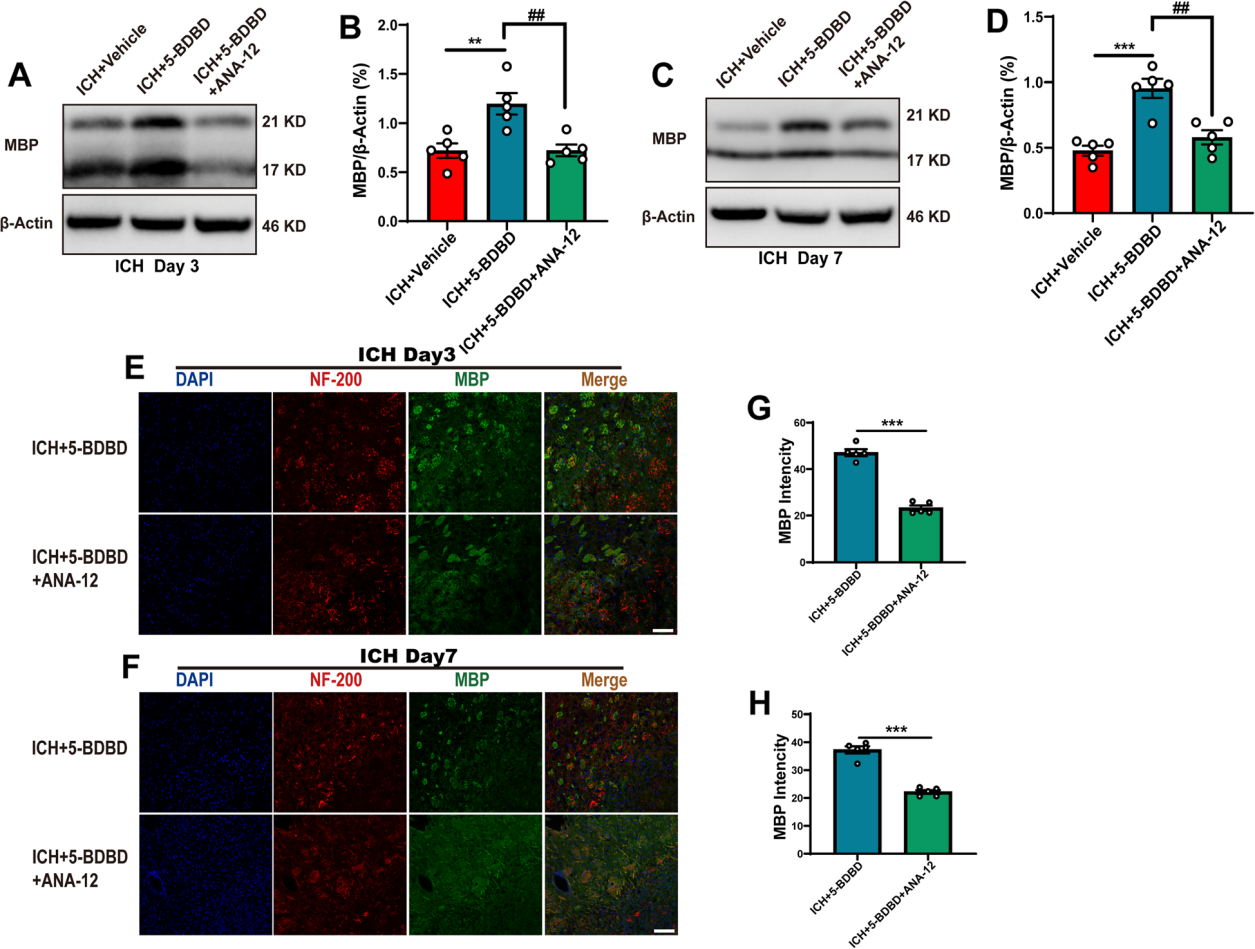
The blockage of TrkB reversed the treatment effect of inhibition P2X4R on white matter injury after ICH. A and B Representative western blot and quantitative analyses of the protein levels of MBP 3 days after ICH (*n* = 5 per group). C and D Representative western blot and quantitative analyses of the protein levels of MBP 7 days after ICH (*n* = 5 per group). E and F Immunostaining for NF200 and MBP in the peri-hematomal region in the ICH + 5-BDBD and ICH + 5-BDBD + ANA-12 groups 3 days and 7 days after ICH. G and H The mean fluorescence densities of MBP in the ICH + 5-BDBD and ICH + 5-BDBD + ANA-12 groups 3 days and 7 days after ICH (*n* = 5 per group). Data are expressed as the means ± SEM. *P < 0.05. **P < 0.01. ***P < 0.001 vs. ICH + 5-BDBD group. ^#^*P* < 0.05. ^##^*P* < 0.01. ^###^*P* < 0.001 vs. ICH + 5-BDBD + ANA-12 group. Scale bar = 100 μm

To investigate whether the inhibition of P2X4R exerts a protective effect on WMI that is dependent on TrkB receptors, we inhibited the activation of TrkB via ANA-12 after 5-BDBD treatment. Our results indicated that, compared with the ICH + 5-BDBD group, the protein level of MBP in the ICH + 5-BDBD + ANA-12 group was significantly decreased 3 and 7 days after ICH (*Fig.* *7**A–D*). Meanwhile, the mean fluorescence intensity of MBP in the ICH + 5-BDBD + ANA-12 group decreased significantly on days 3 and day 7 after ICH as compared with the ICH + 5-BDBD group (*Fig.* *7**E–H*). This suggested that the protective effect of 5-BDBD was counteracted by the administration of ANA-12, an antagonist of TrkB.

**Fig. 8:**
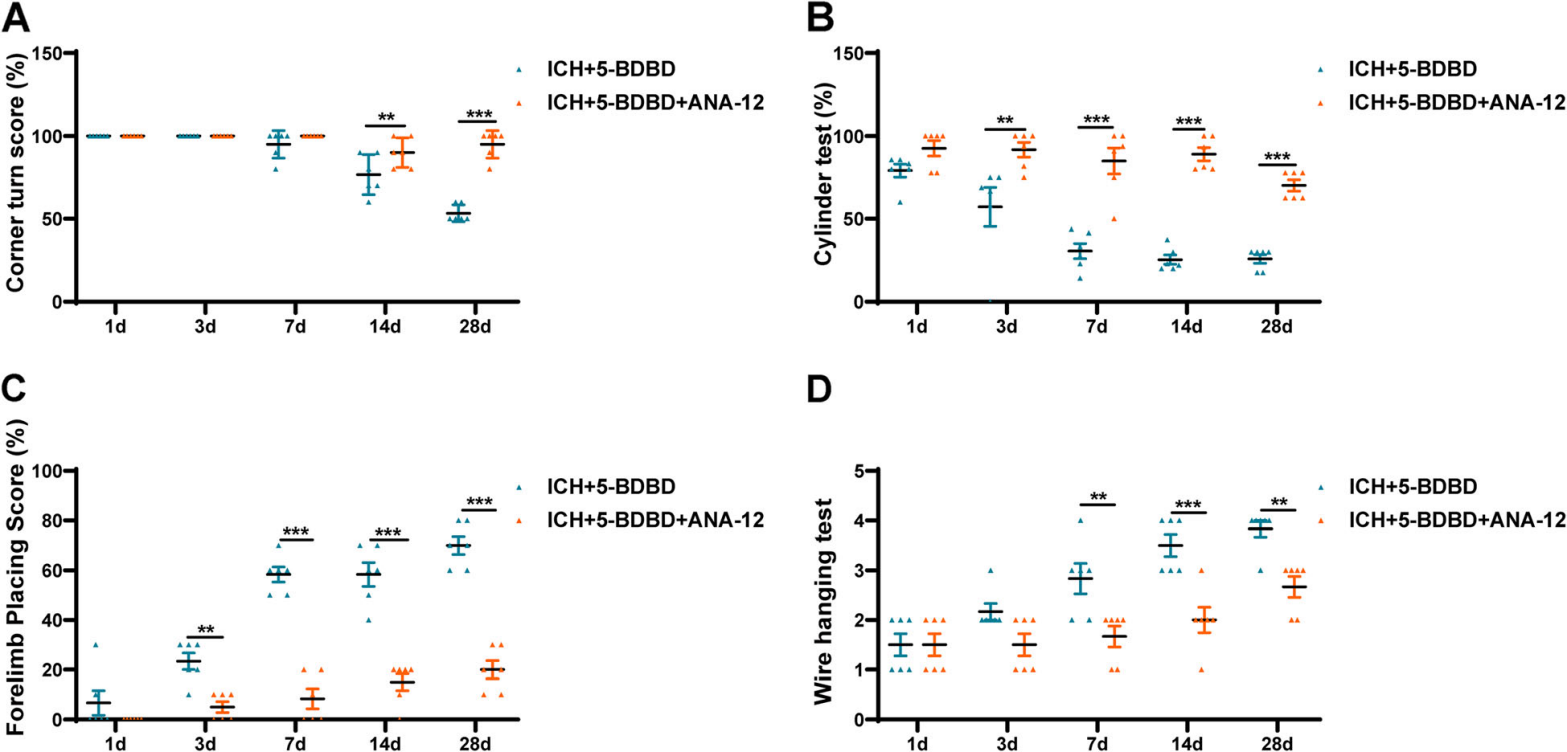
Inhibition of TrkB reversed the treatment effect of inhibition P2X4R on neurological functions after ICH. A Corner turn test, B cylinder test, C forelimb placing test, and D wire hanging test (*n* = 6 per group). Data are expressed as the means ± SEM. *P < 0.05. **P < 0.01. ***P < 0.001 vs. ICH + 5-BDBD group

Meanwhile, we also found that the improvement in neurological function via the administration of 5-BDBD was partly counteracted by ANA-12 (*Fig.* *8*). This suggests that the inhibition of P2X4R may exert a protective effect of WMI that is dependent on the BDNF/TrkB pathway and that this can ameliorate WMI and improve neurological function outcomes.

## Discussion

**Fig. 9:**
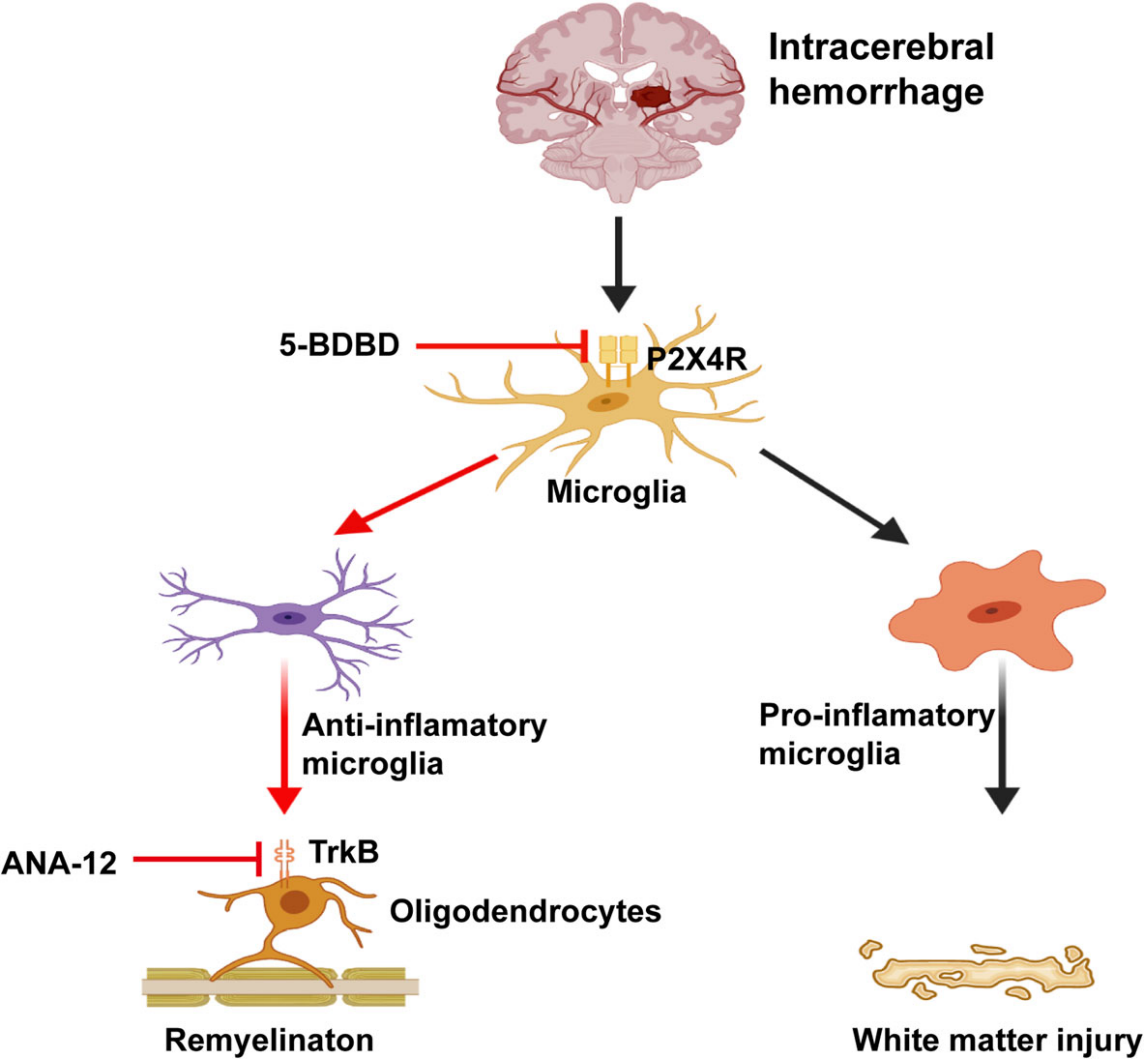
Schematic mechanism of the regulation of microglia P2X4R in neuroinflammation and white matter injury in ICH. The level of P2X4R was upregulated after ICH, leading to the phenotype of microglial polarization into pro-inflammatory. The pharmacological inhibition of P2X4R promoted transformation on microglial phenotypes into anti-inflammatory phenotype and the generation of brain-derived neurotrophic factor, and alleviated white matter injury induced by ICH through the BDNF/TrkB pathway (created with BioRender.com)

In the present study, we identified a key role of P2X4R in protecting WM and mediating neuroinflammation via regulating the microglial phenotypes in a mouse model of ICH. First, our data show that P2X4R is upregulated after ICH, especially on day 7 after ICH and is mainly located in the microglia. Second, inhibition of P2X4R could promote neurological functional recovery after ICH. Third, blockage of P2X4R can promote the transformation of pro-inflammatory microglia induced by ICH into an anti-inflammatory phenotype. Fourth, ICH-induced WMI could be reversed by administration of a P2X4R antagonist. Furthermore, blockage of TrkB can reverse the protective effect of WMI after 5-BDBD treatment. Thus, we conclude that P2X4R plays a crucial role in regulating WMI and neuroinflammation and that P2X4R inhibition may benefit patients with ICH (*Fig.* *9*).

Previous studies have indicated that the immune–inflammatory cascade response exacerbates brain injury after ICH [2, 39, 40]. Microglia are resident myeloid cells in the CNS and can rapidly activate and polarize into a pro-inflammation phenotype after ICH [41, 42]. After acute brain injury, damaged central nerve cells release a large amount of ATP, which causes excessive activation of P2X4R. This activates microglia and aggravates the neuroinflammatory response [21, 43, 44].

P2X4R is a member of the P2X purine receptor family and is highly expressed in activated microglia. This protein has been extensively studied in nervous system diseases [20, 45–47]. Recently, some studies have shown that P2X4R is involved in the regulation of neuroinflammation in ischemic stroke and multiple sclerosis (MS) [21, 47]. After ischemic stroke, the levels of P2X4R in mice increased and that pharmacologically inhibit P2X4R, reduced inflammation, and improved functional recovery. In contrast, in mouse models of MS, P2X4R blockage exacerbates the inflammatory response and P2X4R activation ameliorates neuoinflammation by regulating microglial phenotypes. However, to date, little is known about the function of P2X4R in ICH-induced neuroinflammation; therefore, we explored these functions in an ICH mouse model in the current study.

In our study, we found that after ICH, the protein and transcript levels of P2X4R were significantly elevated, and reaching a peak at day 7. These data suggest that P2X4R is involved in the pathogenesis. In the CNS, P2X4R has been reported to be highly expressed in activated microglia, indicating that P2X4R may be involved in the inflammatory process [48]. Consistent with previous research, our immunofluorescence staining revealed that P2X4R is mainly expressed in activated microglia in the peri-hematoma region. Therefore, we hypothesized that P2X4R is an important regulator of ICH-induced neuroinflammation.

After ICH, the microglia were significantly activated and polarized into a pro-inflammatory phenotype, with increased levels of CD16/32 (pro-inflammatory microglia marker). Our data indicated that the inhibition of P2X4R with 5-BDBD could significantly attenuate the inflammatory response by shifting the phenotype toward an anti-inflammatory phenotype after ICH. This was confirmed by the observed decreased expression levels of CD16/32, IL-1β, and TNF-α, as well as increased levels of the anti-inflammatory microglia marker Arg-1 and CD206. This result suggests that P2X4R activation regulates microglial polarization and enhances neuroinflammation after ICH. Pharmacological inhibition of P2X4R significantly alleviated ICH-induced neuroinflammation and secondary brain injury by reducing the phenotype of pro-inflammatory microglia and increasing anti-inflammatory microglia. However, based on our results and the results of previous studies, the detailed mechanisms underlying P2X4R control of microglia polarization require further study.

The predilection sites of ICH are located in the basal ganglia, the internal capsule, and the thalamus, which are rich in white matter fibers [13]. This indicates that WMI may be a dominant type of brain injury following ICH [49]. Hence, we explored the therapeutic effect of inhibiting P2X4R with 5-BDBD on WMI. First, we found that ICH induced severe WMI. Moreover, we found that the inhibition of P2X4R with 5-BDBD can significantly attenuate the injury of WM fibers after ICH, thereby contributing to improved neurological function. WMI is a key factor in ICH patient outcomes. A previous study found that ICH-induced neuroinflammation is a major cause of WMI [10, 50]. Targeting microglia, a major cell type in ICH-induced neuroinflammation may be an effective therapeutic strategy for WMI.

Previous reports have demonstrated that anti-inflammatory microglia can enhance BDNF production, promote axon sprouting and preserve myelin integrity [12, 51, 52]. In line with a previous study, our data showed that the inhibition of P2X4R with 5-BDBD can significantly increase BDNF levels by promoting the microglial phenotype to an anti-inflammatory phenotype after ICH. Therefore, we hypothesized that the inhibition of P2X4R attenuates WMI after ICH in mice by regulating the microglial phenotypes.

Next, we investigated the underlying mechanism of P2X4R in WMI after ICH. Oligodendrocytes play a key role in maintaining WM homeostasis [53]. TrkB, the BDNF receptor that is expressed in oligodendrocytes and combined with BNDF, can promote remyelination [47]. Consistently, our data showed that TrkB is found in oligodendrocytes. TrkB is located directly downstream of BDNF, and previous studies have shown that activated TrkB plays a neuroprotective function after ischemic stroke and traumatic brain injury [53, 54]. The TrkB inhibitor (ANA-12) was applied to ICH mice in our study. Treatment with the TrkB inhibitor significantly reversed the effects of 5-BDBD in attenuated WMI and decreased MBP expression levels. The effect of improved neurological outcomes after 5-BDBD treatment was also partially reversed by ANA-12 treatment. These data further support the role of P2X4R in regulating WMI after ICH through the BNDF/TrkB pathway. Our results indicate that P2X4R promotes neuroinflammation and WMI. The pharmacological inhibition of P2X4R attenuated WMI after ICH, and our results show that this is mediated at least in part by the BDNF/TrkB pathway. The activated BDNF/TrkB pathway exerts neuroprotective effects in many CNS diseases, including acute brain injury and neurodegenerative diseases [55, 56]. However, activation of the BDNF/TrkB pathway plays an opposite role in epilepsy. In animal models and patients with epilepsy, both BDNF and TrkB are increased, and BDNF injected into the brain induces seizures. Current evidence indicates that inhibiting the BDNF/TrkB pathway is a potential therapeutic strategy for epilepsy [57, 58]. This pathway should be investigated more thoroughly in future studies.

P2X7R is another member of the P2X purine receptor family and is also highly expressed in activated microglia. The function and mechanisms of P2X7R in ICH-induced acute brain injury have been widely discussed. On one hand, it has been clarified that P2X7R contributes to ICH-induced neuroinflammation. After ICH, the level of P2X7R was significantly elevated and directly interacted with the NLRP3 inflammasome, promoting neuroinflammatory progression via IL-1β release and neutrophil infiltration [59, 60]. On the other hand, after ICH, the increased P2X7R aggravated NOX2-induced oxidative stress through the activation of the ERK1/2 and NF-κB pathways [61]. Moreover, P2X4R might be able to form heteromers with P2X7R [62]. However, to date, the function of P2X7R in ICH-induced WMI is unclear. Consistent with previous research, our results revealed that P2X7R is upregulated after ICH. The increasing trend was mostly not affected by 5-BDDB 3 and 7 days after ICH. Based on this data, we were not able to confirm the effect of P2X7R in this process of ICH-induced WMI. However, in a future study, we will attempt to explore the function and mechanism of P2X7R in WMI after ICH and explore the relationship between P2X4R and P2X7R after ICH.

Although our research has provided evidence that inhibition of P2X4R attenuates WMI by regulating microglial phenotypes and improves the outcomes in ICH male mouse models, there are several limitations to our study. First, it is possible that microglia P2X4R may act with other potent receptors in the ICH-induced immune–inflammatory responses. Further research is required to explore other possible associations mediated by P2X4R after ICH. Second, we investigated the impact and mechanisms of P2X4R in male mice. However, estrogen levels and sex may affect ICH outcomes [63]. Therefore, further research is necessary to determine the function of P2X4R in female mice after ICH. Third, age is an important factor that affects the functional outcomes of many diseases. However, in the present study, we only focused on the role of P2X4R in young mice; therefore, more research is necessary to explore the function and underlying mechanisms of P2X4R in older ICH mouse models. Moreover, in the present research, we only focused on the short-term effects of P2X4R inhibition. The long-term effect of P2X4R inhibition in ischemic stroke is controversial [21, 64]; therefore, these long-term effects need to be further explored after ICH. In addition, our research pharmacologically inhibited only P2X4R and TrkB, though these inhibitors are widely used, and their effectiveness has been tested in prior research. However, the use of AAV virus or knockout mice would improve the quality of our research.

## Conclusions

Our results demonstrated that inhibition of P2X4R promoted the pro-inflammatory microglia polarization into an anti-inflammatory phenotype and enhanced BDNF production, as well as alleviating WMI and improving neurological outcomes through the BDNF/TrkB pathway. Therefore, regulation of P2X4R activation may be beneficial for reducing ICH-induced brain injury.

## Supplementary Information

**Additional file 1.** Figure S1. The experimental design schematic, drug dosages, and animal groups (Part figure was created with BioRender.com).

**Additional file 2.** Figure S2. The general schematic diagram of ROI for immunostaining images.

**Additional file 3 **Figure S3. A. qPCR analysis of genes encoding transcription of P2X4R at different time points after ICH. (n = 3). B. Quantification of P2X4R^+^ cells. (n = 3). Data are expressed as the means ± SEM. *P<0.05. **P<0.01. ***P<0.001. Scale bar = 100μm.

**Additional file 4.** Figure S4.The optimal dose for 5-BDBD in ICH mice.

**Additional file 5 **Figure S5. A - B. The levels of mRNA transcription of IL-1β, TNF-α, and CD206 were examined by PCR in sham, ICH + vehicle, and ICH + 5-BDBD groups after ICH day 3 and day 7. (n = 3 per group). C - D. Microglia counts and morphology were analyzed after 5-BDBD treatment in ICH mice (n = 5 per group). E. The Schematic diagram of microglia morphology in ICH + vehicle and ICH + 5-BDBD groups. F – H. The data of microglia soma area, endpoints, and process length in ICH + vehicle and ICH + 5-BDBD groups. *P<0.05. **P<0.01. ***P<0.001 vs. ICH + vehicle group. ^#^P<0.05. ^##^P<0.01. ^###^P<0.001 vs. ICH + 5-BDBD group. Scale bar = 50μm.

**Additional file 6 **Figure S6. A - B. Representative Western blot and quantitative analyses of the protein levels of P2X7R in the 5-BDBD treatment groups compared with the vehicle group 7 days after ICH. (n = 3 per group, two repetitions). C. The levels of mRNA transcription of P2X7R was examined by PCR in sham, ICH + vehicle, and ICH + 5-BDBD groups after ICH day 7. (n = 3 per group).Data are expressed as the means ± SEM. *P<0.05. **P<0.01. ***P<0.001 vs. ICH + vehicle group.

**Additional file 7.** Figure S7. Representative images of co-localization of TrkB (green) with oligodendrocytes (Olig2, red) in the perihematomal region. Scale bar =50 μm.

**Additional file 8 **Figure S8. The effect of ANA-12 alone treatment on WMI after ICH and the effect of ANA-12 on TrkB activities after ICH. A - B. Representative Western blot and quantitative analyses of the protein levels of MBP 3 days after ICH. (n = 3 per group, two repetitions). C - D. Representative Western blot and quantitative analyses of the protein levels of MBP 7 days after ICH. (n = 3 per group, two repetitions). E - G. Immunostaining MBP in the peri-hematomal region in the ICH + vehicle and ICH+ ANA-12 groups 3 and 7 days after ICH. F - H. Mean fluorescence densities of MBP in the ICH + vehicle and ICH+ ANA-12 groups 3 and 7 days after ICH. (n = 5 per group). I. Representative Western blot and quantitative analyses of the protein levels of p-TrkB and TrkB 7 days after ICH. Data are expressed as the means ± SEM. *P<0.05. **P<0.01. ***P<0.001 vs. ICH + vehicle group. ^#^P<0.05. ^##^P<0.01. ^###^P<0.001 vs. ICH + ANA-12 group. Scale bar = 100μm.

**Additional file 9.** Table S1. Primers used in RT-PCR.

## Data Availability

All raw data in this research are available on reasonable request.

## Abbreviations

APP: Amyloid precursor protein
BDNF: Brain-derived neurotrophic factor
CNS: Central nervous system
cDNA: Complementary deoxyribonucleic acid
GFAP: Glial fibrillary acidic protein
ICH: Intracerebral hemorrhage
Iba-1: Ionized calcium binding adaptor molecule
MBP: Myelin basic protein
NeuN: Neuronal specific nuclear protein
NF-200: Neurofilament-200
PFA: Paraformaldehyde
PBS: Phosphate-buffered saline
P2X4R: P2X purinoreceptor 4
P2X7R: P2X purinoreceptor 7
qPCR: Quantitative polymerase chain reaction
RTK: Receptor tyrosine kinase
ROIs: Regions of interest
SPF: Specific pathogen-free
SME: Standard error of the mean
TrkB: Tropomyosin-related kinase receptor B
WMI: White matter injury
WM: White matter
WB: Western blots
