# Supplementary figures and images for: Inhibition of P2X4R attenuates white matter injury in mice after intracerebral hemorrhage by regulating microglial phenotypes
Source: J Neuroinflammation. 2021 Aug 23;18:184. doi: 10.1186/s12974-021-02239-3 (PMC8383380; doi:10.1186/s12974-021-02239-3)

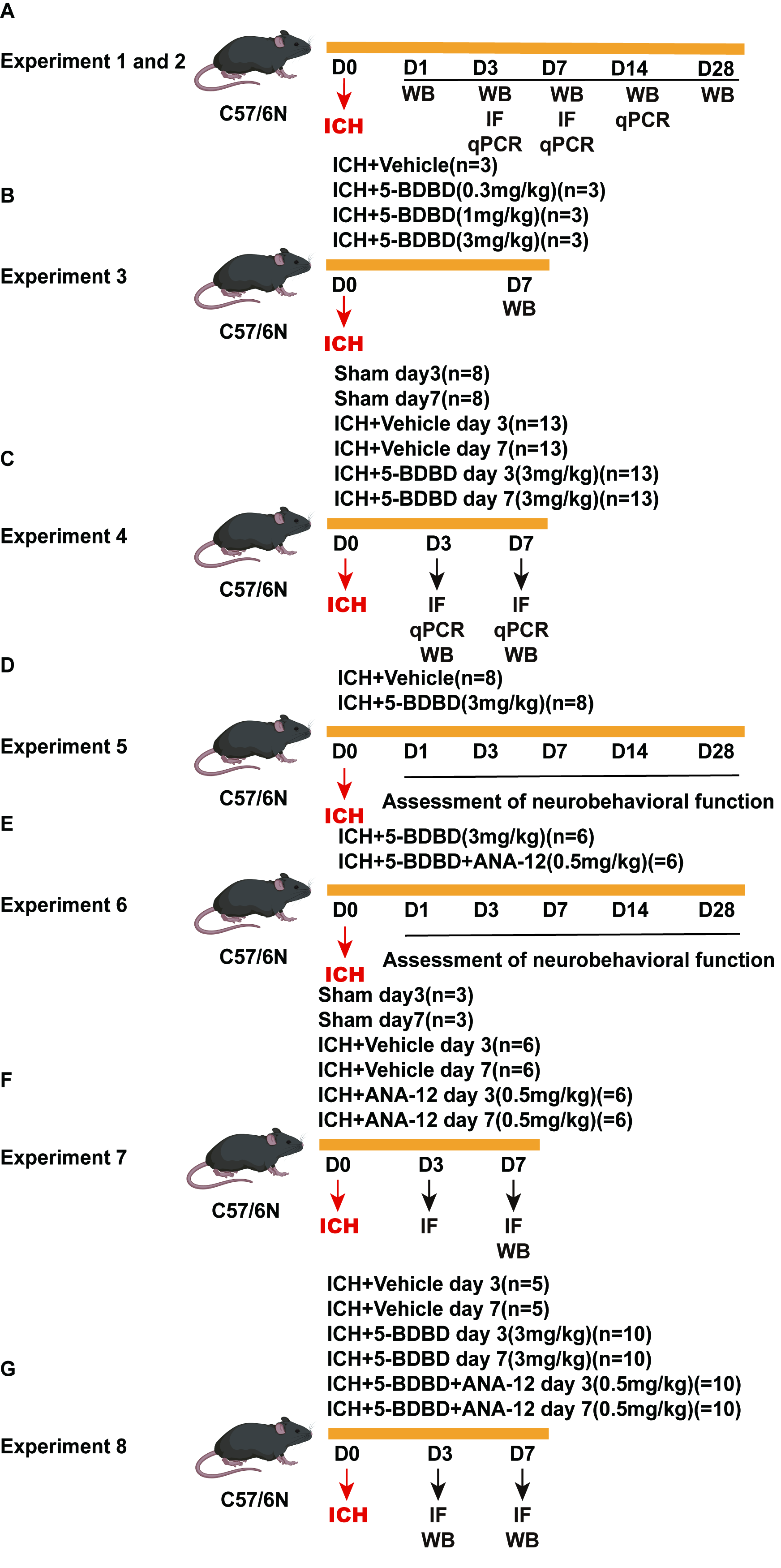

Supplement: Supplementary file 1 — Additional file 1. Figure S1. The experimental design schematic, drug dosages, and animal groups (Part figure was created with BioRender.com). [file 12974_2021_2239_MOESM1_ESM.tif]

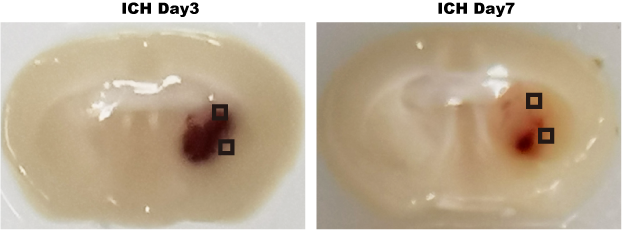

Supplement: Supplementary file 2 — Additional file 2. Figure S2. The general schematic diagram of ROI for immunostaining images. [file 12974_2021_2239_MOESM2_ESM.tif]

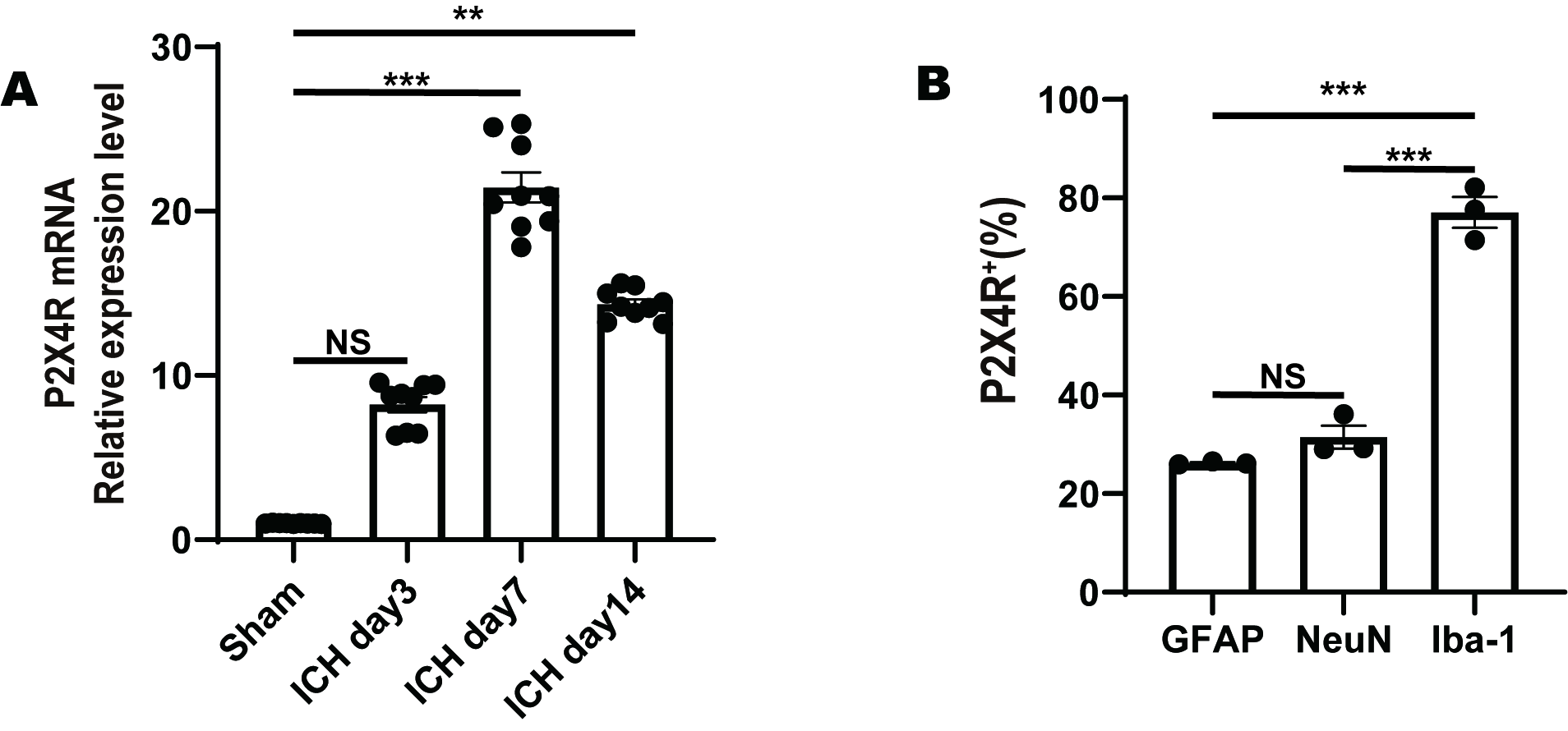

Supplement: Supplementary file 3 — Additional file 3 Figure S3. A. qPCR analysis of genes encoding transcription of P2X4R at different time points after ICH. (n = 3). B. Quantification of P2X4R+ cells. (n = 3). Data are expressed as the means ± SEM. *P<0.05. **P<0.01. ***P<0.001. Scale bar = 100μm. [file 12974_2021_2239_MOESM3_ESM.tif]

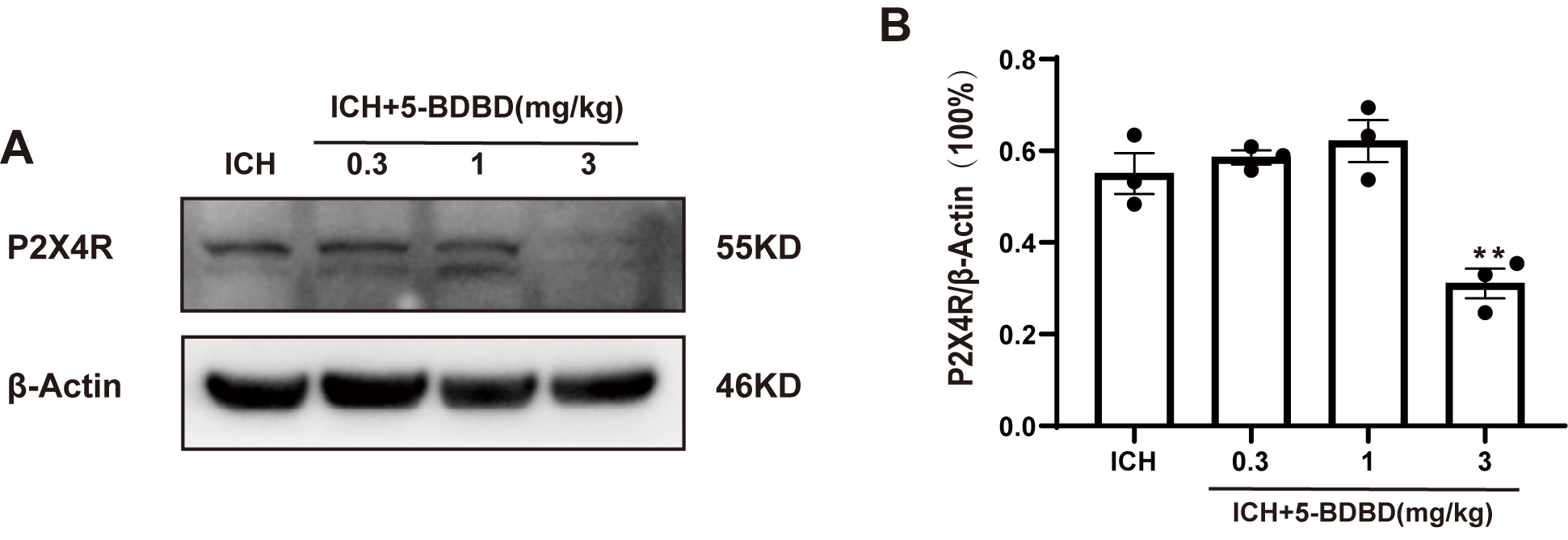

Supplement: Supplementary file 4 — Additional file 4. Figure S4.The optimal dose for 5-BDBD in ICH mice. [file 12974_2021_2239_MOESM4_ESM.tif]

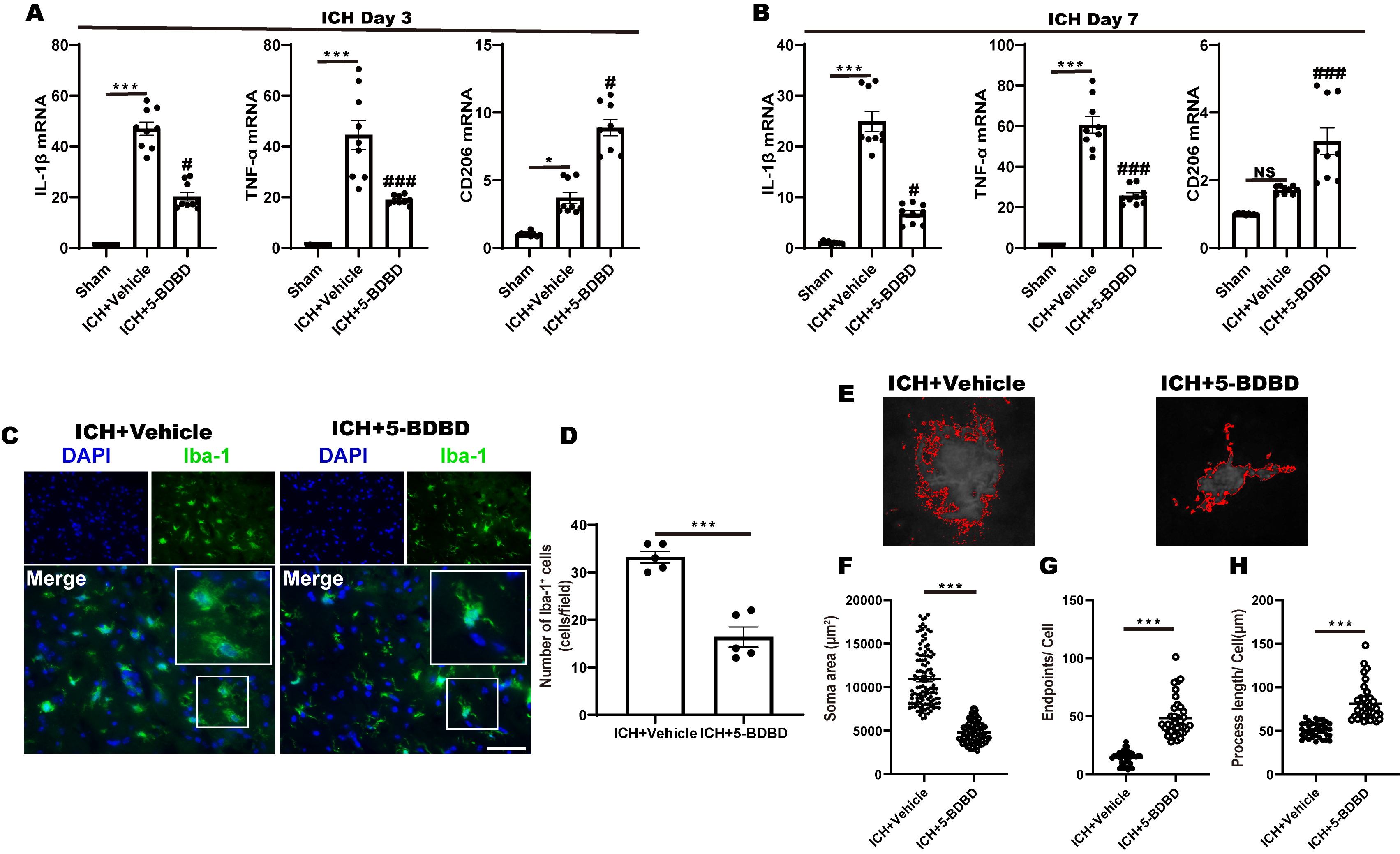

Supplement: Supplementary file 5 — Additional file 5 Figure S5. A - B. The levels of mRNA transcription of IL-1β, TNF-α, and CD206 were examined by PCR in sham, ICH + vehicle, and ICH + 5-BDBD groups after ICH day 3 and day 7. (n = 3 per group). C - D. Microglia counts and morphology were analyzed after 5-BDBD treatment in ICH mice (n = 5 per group). E. The Schematic diagram of microglia morphology in ICH + vehicle and ICH + 5-BDBD groups. F – H. The data of microglia soma area, endpoints, and process length in ICH + vehicle and ICH + 5-BDBD groups. *P<0.05. **P<0.01. ***P<0.001 vs. ICH + vehicle group. #P<0.05. ##P<0.01. ###P<0.001 vs. ICH + 5-BDBD group. Scale bar = 50μm. [file 12974_2021_2239_MOESM5_ESM.tif]

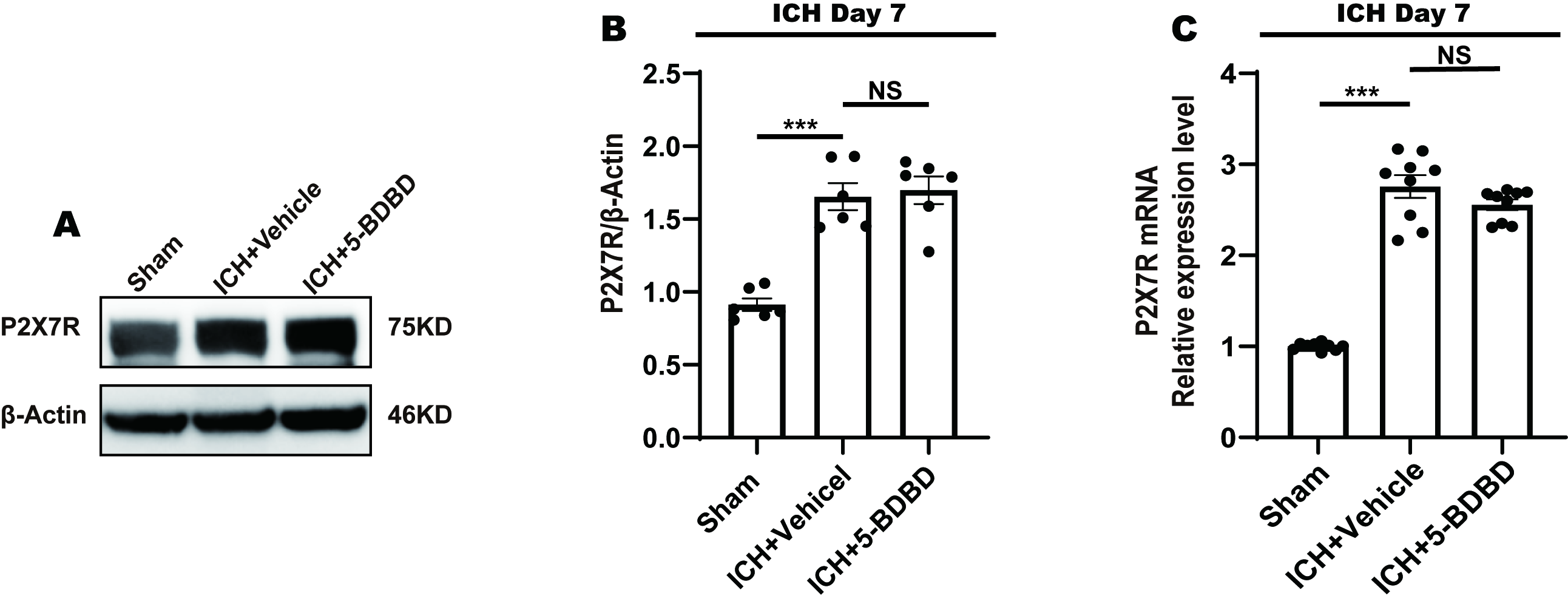

Supplement: Supplementary file 6 — Additional file 6 Figure S6. A - B. Representative Western blot and quantitative analyses of the protein levels of P2X7R in the 5-BDBD treatment groups compared with the vehicle group 7 days after ICH. (n = 3 per group, two repetitions). C. The levels of mRNA transcription of P2X7R was examined by PCR in sham, ICH + vehicle, and ICH + 5-BDBD groups after ICH day 7. (n = 3 per group).Data are expressed as the means ± SEM. *P<0.05. **P<0.01. ***P<0.001 vs. ICH + vehicle group. [file 12974_2021_2239_MOESM6_ESM.tif]

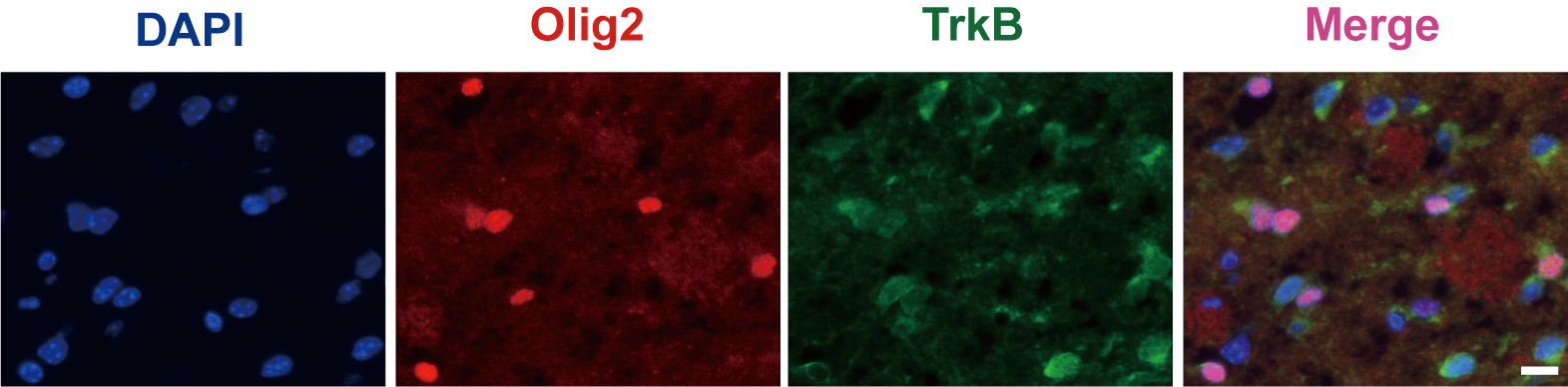

Supplement: Supplementary file 7 — Additional file 7. Figure S7. Representative images of co-localization of TrkB (green) with oligodendrocytes (Olig2, red) in the perihematomal region. Scale bar =50 μm. [file 12974_2021_2239_MOESM7_ESM.tif]

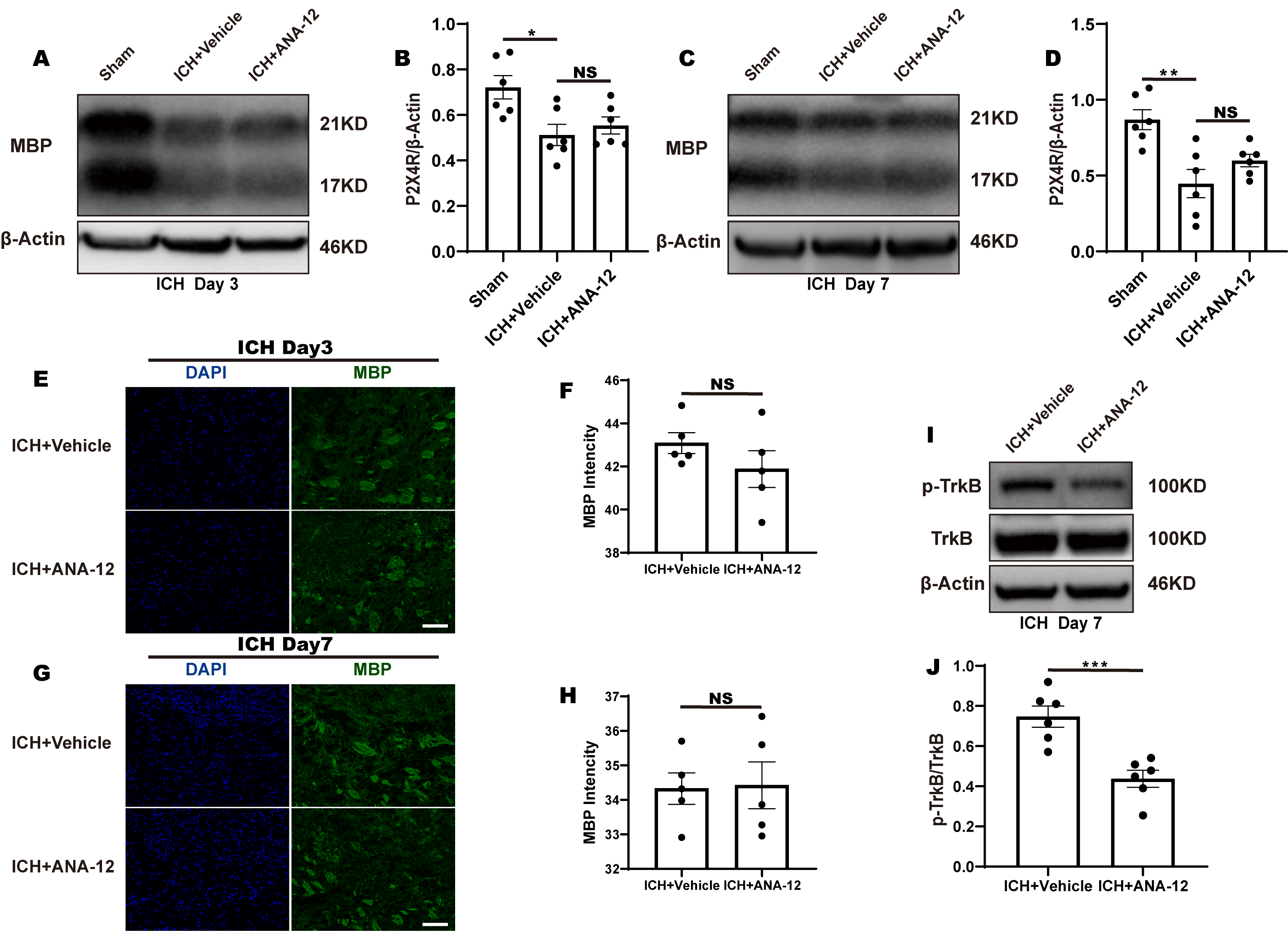

Supplement: Supplementary file 8 — Additional file 8 Figure S8. The effect of ANA-12 alone treatment on WMI after ICH and the effect of ANA-12 on TrkB activities after ICH. A - B. Representative Western blot and quantitative analyses of the protein levels of MBP 3 days after ICH. (n = 3 per group, two repetitions). C - D. Representative Western blot and quantitative analyses of the protein levels of MBP 7 days after ICH. (n = 3 per group, two repetitions). E - G. Immunostaining MBP in the peri-hematomal region in the ICH + vehicle and ICH+ ANA-12 groups 3 and 7 days after ICH. F - H. Mean fluorescence densities of MBP in the ICH + vehicle and ICH+ ANA-12 groups 3 and 7 days after ICH. (n = 5 per group). I. Representative Western blot and quantitative analyses of the protein levels of p-TrkB and TrkB 7 days after ICH. Data are expressed as the means ± SEM. *P<0.05. **P<0.01. ***P<0.001 vs. ICH + vehicle group. #P<0.05. ##P<0.01. ###P<0.001 vs. ICH + ANA-12 group. Scale bar = 100μm. [file 12974_2021_2239_MOESM8_ESM.tif]
