# Supplementary material for: Inhibition of P2X4R attenuates white matter injury in mice after intracerebral hemorrhage by regulating microglial phenotypes
Source: J Neuroinflammation. 2021 Aug 23;18:184. doi: 10.1186/s12974-021-02239-3 (PMC8383380; doi:10.1186/s12974-021-02239-3)
Supplement: Supplementary file 9 — Additional file 9. Table S1. Primers used in RT-PCR. [file 12974_2021_2239_MOESM9_ESM.docx]

**Table S1** Primers used in RT-PCR

| Primer sequences(5^,^-3^,^) | | |
| --- | --- | --- |
| Gene | Forward | Reverse |
| P2X4R | TTTGCGATTCAGACGCCAAC | ATGGAACACACCTTCCAGTCC |
| CD16/32 | TTTGGACACCCAGATGTTTCAG | GTCTTCCTTGAGCACCTGGATC |
| Arg-1 | CGCCTTTCTCAAAAGGACAG | CCAGCTCTTCATTGGCTTTC |
| IL-1β | CAACCAACAAGTGATATTCTCCATG | GATCCACACTCTCCAGCTGCA |
| TNF-α | ATGGCCTCCCTCTCAGTTC | TTGGTGGTTTGCTACGACGTG |
| CD206 | CAAGGAAGGTTGGCATTTGT | CCTTTCAGTCCTTTGCAAGC |
| P2X7R | AGCACGAATTATGGCACCGT | CCCCACCCTCTGTGACATTCT |
| β-Actin | AGGCATTGTGATGGACTCCG | AGCTCAGTAACAGTCCGCCTA |
